# Long‐term abundance time‐series of the High Arctic terrestrial vertebrate community of Bylot Island, Nunavut

**DOI:** 10.1002/ecy.70223

**Published:** 2025-10-07

**Authors:** Louis Moisan, Azenor Bideault, Gilles Gauthier, Éliane Duchesne, Dominique Fauteux, Dominique Berteaux, Pierre Legagneux, Marie‐Christine Cadieux, Joël Bêty

**Affiliations:** ^1^ Chaire de Recherche du Canada en Biodiversité Nordique, Centre d'Études Nordiques, Centre de la Science de la Biodiversité du Québec, Département de Biologie, Chimie et Géographie Université du Québec à Rimouski Rimouski Quebec Canada; ^2^ Chaire de Recherche du Canada en Écologie Intégrative, Centre d'Études Nordiques, Centre de la Science de la Biodiversité du Québec, Département de Biologie Université de Sherbrooke Sherbrooke Quebec Canada; ^3^ Chaire de Recherche Sentinelle Nord sur l'Impact des Migrations Animales sur les Écosystèmes Nordiques, Centre d'Études Nordiques, Centre de la Science de la Biodiversité du Québec, Département de Biologie Université Laval Québec Quebec Canada; ^4^ Centre d'Études Nordiques, Département de Biologie Université Laval Québec Quebec Canada; ^5^ Centre d'Études Nordiques, Centre de Connaissance et d'Exploration de l'Arctique, Musée Canadien de la Nature Ottawa Ontario Canada; ^6^ Centre d'Études Biologiques de Chizé (CEBC‐CNRS), Université de La Rochelle Nouvelle‐Aquitaine France

**Keywords:** Arctic tundra, biodiversity monitoring, Bylot Island, Canadian Arctic, community composition, community structure, food web, long‐term monitoring, species abundance, species biomass, species body mass

## Abstract

Arctic ecosystems present unique opportunities for community‐wide monitoring, in part due to their relatively low species richness. However, conducting research in these remote environments poses significant logistical challenges, resulting in long‐term monitoring being exceedingly rare. Here, we focus on the long‐term, intensive ecological monitoring efforts conducted on the south plain of Bylot Island (~400 km^2^, Nunavut, Canada), which has generated a remarkable dataset spanning up to 30 years, a rarity in tundra ecosystems. Our goals are to (1) provide long‐term time‐series of annual vertebrate density measured at various spatial scales and for the broadest possible range of species and years, to allow the assessment of interannual variability and trends in species density; and (2) upscale annual vertebrate abundance or sometimes long‐term averages to the landscape scale (400 km^2^) to allow food web modeling. Monitoring data include intensive capture–mark–recapture density estimates of lemmings on trapping grids, systematic or opportunistic nest monitoring conducted across the entire study area or within specific plots for all bird species, transects of vertebrate counts distributed throughout the study area, daily incidental observations of vertebrates, and satellite tracking of foxes. We standardized data obtained with different field methods to provide a readily usable dataset for community ecologists. Long‐term time‐series of vertebrate densities span 3–27 years, with a median of 16.5 years for 22 species. We estimated landscape‐scale abundance for all 35 species of the community based on annual time‐series for 15 of them and average abundance for the remaining 20 species. Furthermore, we provide body mass data for each species, based on empirical onsite measurements for 18 species and from the literature for the remaining species. Body mass is essential to convert species abundance into biomass for studies of trophic fluxes and ecosystem processes. Daily climatic data recorded since 1992 from weather stations within the study area are also available and complement the vertebrate dataset. The ecological data presented offer a rare opportunity for holistic empirical studies of community structure and dynamics. Considering that the study site is a pristine and protected area that has experienced minimal direct anthropogenic impact, it also provides an ideal baseline for investigating the impacts of global changes on high‐latitude terrestrial ecosystems. There are no copyright restrictions on the data or code, and this data paper should be cited when these items are reused.

## CONFLICT OF INTEREST STATEMENT

The authors declare no conflicts of interest.

## Supporting information


Data S1:

## Data Availability

The complete dataset is available in Data S1 as Supporting Information. The complete dataset, including raw data, is also archived in Dryad at https://doi.org/10.5061/dryad.44j0zpcnt. Comprehensive documentation on the study context, objectives, methods, and metadata is archived in Zenodo at https://doi.org/10.5281/zenodo.16794620. The code and the complete R project used to estimate species abundance are archived in Zenodo at https://doi.org/10.5281/zenodo.16794619. Moreover, long‐term climatic data within the study area are available in the NordicanaD repository at https://doi.org/10.5885/45039SL-EE76C1BDAADC4890. Finally, raw monitoring data for the following key species of the food web are also available at the NordicanaD data repository (https://nordicana.cen.ulaval.ca/en/list-of-publications.php) and are periodically updated as the field studies continue on Bylot Island: Lemming monitoring on Bylot Island; Monitoring of Greater Snow Goose reproduction on Bylot Island; Monitoring of Lapland longspur reproduction on Bylot Island; Monitoring of shorebirds reproduction on Bylot Island; Monitoring of arctic and red fox reproduction on Bylot Island; Monitoring of avian predator reproduction on Bylot Island; Relative abundance of tundra bird and mammal species encountered daily on Bylot Island.

