# Supplementary material for: Long‐term abundance time‐series of the High Arctic terrestrial vertebrate community of Bylot Island, Nunavut
Source: Ecology. 2025 Oct 7;106(10):e70223. doi: 10.1002/ecy.70223 (PMC12501677; doi:10.1002/ecy.70223)
Supplement: Supplementary file 1 — Data S1: [file ECY-106-e70223-s001.zip › Data_S1/README.html]

README


# Long-term abundance time-series of the High Arctic terrestrial vertebrate community of Bylot Island, Nunavut

### **Access data, metadata and code**

#### Data - `Data_S1/`: https://doi.org/10.5061/dryad.44j0zpcnt

#### Methods and metadata - `MetadataS1.pdf`: https://doi.org/10.5281/zenodo.13993828

#### Code and R project - `BYLOT_species_abundance_dataset.zip`: https://doi.org/10.5281/zenodo.13993826

### **Abstract**

Arctic ecosystems present unique opportunities for community-wide
monitoring, in part due to their relatively low species richness.
However, conducting research in these remote environments poses
significant logistical challenges, resulting in long-term monitoring
being exceedingly rare. Here, we focus on the long-term, intensive
ecological monitoring efforts conducted on the south plain of Bylot
Island (~400 km², Nunavut, Canada), which has generated a remarkable
dataset spanning up to 30 years, a rarity in tundra ecosystems. Our
goals are to i) provide long-term time series of annual vertebrate
density measured at various spatial scales and for the broadest possible
range of species and years, to allow the assessment of interannual
variability and trends in species density; and ii) upscale annual
vertebrate abundance or sometimes long-term averages to the landscape
scale (400 km²) to allow food web modelling. Monitoring data includes
intensive capture-mark-recapture density estimates of lemmings on
trapping grids, systematic or opportunistic nest monitoring conducted
across the entire study area or within specific plots for all bird
species, transects of vertebrate counts distributed throughout the study
area, daily incidental observations of vertebrates and satellite
tracking of foxes. We standardized data obtained with different field
methods to provide a readily usable data set for community ecologists.
Long-term time series of vertebrate densities span 3 to 27 years, with a
median of 16.5 years for 22 species. We estimated landscape-scale
abundance for all 35 species of the community based on annual time
series for 15 of them and average abundance for the remaining 20
species. Furthermore, we provide body mass data for each species, based
on empirical onsite measurements for 18 species and from the literature
for the remaining species. Body mass is essential to convert species
abundance into biomass for studies of trophic fluxes and ecosystem
processes. Daily climatic data recorded since 1992 from weather stations
within the study area are also available and complement the vertebrate
dataset. The ecological data presented offer a rare opportunity for
holistic empirical studies of community structure and dynamics.
Considering that the study site is a pristine and protected area that
has experienced minimal direct anthropogenic impact, it also provides an
ideal baseline for investigating the impacts of global changes on
high-latitude terrestrial ecosystems.

### **Objectives**

Our goals are to i) provide long-term time series of annual
vertebrate density measured at various spatial scales and for the
broadest possible range of species and years, to allow the assessment of
interannual variability and trends in species density; and ii) upscale
annual vertebrate abundance or sometimes long-term averages to the
landscape scale (400 km²) to allow food web modelling (Figure 2). We
focus on the breeding season (May to August) here due to the absence of
monitoring during the non-breeding season; however, we note that
ecological dynamics during the non-breeding period, for both resident
(Hutchison et al., 2020) and migratory species (Moisan et al., 2023),
can influence the food web during the breeding season. The estimates
include both breeding and non-breeding individuals that remain in the
study area for a significant period of time (e.g., territorial breeding
or non-breeding foxes), and excludes non-breeding individuals that stop
only briefly during migration (e.g., shorebirds using Bylot Island for a
short stopover). We focus on adults, except for lemmings for which we
have not distinguished between juveniles and adults. Additionally, we
aim to provide the average body mass for each species in the community,
enabling the conversion of abundances into biomasses.

### **Structure of the project**

#### **Data\_S1/**

> **`BYLOT-species_taxonomy.csv`**: Species taxonomy.

- *class*: Taxonomic class for birds (Gill et al., 2024) and
  mammals species (Upham et al., 2024).
- *order*: Taxonomic order for birds (Gill et al., 2024) and
  mammals species (Upham et al., 2024).
- *family*: Taxonomic family for birds (Gill et al., 2024) and
  mammals species (Upham et al., 2024).
- *genus*: Taxonomic genus for birds (Gill et al., 2024) and
  mammals species (Upham et al., 2024).
- *species\_scientific*: Taxonomic species for birds (Gill et
  al., 2024) and mammals species (Upham et al., 2024).
- *species\_en*: Common names of species in English.
- *species\_fr*: Common names of species in French.
- *functional\_group*: Functional group for each species. The
  classification of species into functional groups is based on Moisan et
  al. (2023).
- *migratory\_status*: Migratory status of each species. The
  classification of species migratory status is based on Gauthier et al.,
  (2011) and Moisan et al. (2023).

> **`BYLOT-species_density_monitoring.csv`**: Annual
> species density measured in the different zones of the study area
> (various spatial scales).

- *species\_en*: Common names of species in English.
- *year*: Year in which species density was monitored in the
  corresponding zone.
- *zone*: Zone of the study area in which species density was
  measured.
- *area\_sampled\_km2*: Area (in square kilometers) sampled to
  measure species density.
- *habitat*: Habitat in which species density was
  measured.
- *method*: Method used to measure species density.
- *monitoring*: Type of monitoring conducted, distinguishing
  between systematic (planned, standardized data collection) and
  opportunistic (irregular or incidental).
- *breeding\_status*: Reproductive status of the
  individuals.
- *ind\_density\_km2*: Density of individuals (number of
  individuals per square kilometer) measured in the corresponding year and
  zone.

> **`BYLOT-species_abundance.csv`**: Long-term series or
> mean species abundance.

- *species\_en*: Common names of species in English.
- *year*: Year corresponding to the estimate of annual
  abundance. If abundance has not been calculated for a given series of
  years, but rather as a general average, then NA has been assigned.
- *breeding\_status*: Reproductive status of the
  individuals.
- *abundance*: Estimate of the number of individuals within the
  389 km2 study area located on the southern part of Bylot Island during
  the breeding season (May to August). The estimates only consider adults,
  with the exception of lemmings, for which no distinction has been made
  between juveniles and adults.
- *method\_description*: Brief overview of the method used to
  estimate the species abundance.
- *method\_quality*: Qualitative measure of the method quality
  based on data available, method used for extrapolation (if necessary),
  and in some cases, from the fit of statistical models to estimate
  density.
- *spatial\_extrapolation*: Indicates whether the abundance
  estimate was derived from monitoring across the entire study area (“no”)
  or was spatially extrapolated (“yes”).

> **`BYLOT-community_composition.csv`**: Estimated annual
> or mean number of individuals for each species in the vertebrate
> community of Bylot Island between May and August. For all species, we
> estimated the abundance of adults, except for lemmings, for which
> juveniles and adults were not distinguished. When multiple independent
> methods were available to estimate the annual or mean abundance of a
> species, we retained the estimate from the method with the highest
> assigned quality. If methods had equal quality, we computed and
> presented the mean of their estimates.

- *species\_en*: Common names of species in English.
- *1993 - 2023*: Estimate of the annual number of individuals
  within the 389 km2 study area located on the southern part of Bylot
  Island during the breeding season (May to August) for the corresponding
  year. The estimates represent only breeding individuals, except for
  lemmings, for which the estimates include both breeding and non-breeding
  individuals. The estimates exclude non-breeding individuals that stop
  for only a few days during their migration. The estimates only consider
  adults, with the exception of lemmings, for which no distinction has
  been made between juveniles and adults. Note that 2020 does not appear
  since fieldwork was not possible due to Covid-19.
- *mean*: Estimate of the average annual number of individuals
  within the 389 km2 study area located on the southern part of Bylot
  Island during the breeding season (May to August). This includes both
  breeding and non-breeding individuals that stay in the study area for a
  significant period of time, and excludes non-breeding individuals that
  stop for only a few days during their migration. The estimates only
  consider adults. For species where abundance was estimated using
  multiple methods, we selected the method determined to be of the highest
  quality. In cases where multiple methods yielded equivalent quality, the
  mean value of their estimates was retained.

> **`BYLOT-species_body_mass.csv`**: Mean individual body
> mass.

- *species\_en*:Common names of species in English.
- *site*: Site where body mass measurements were taken.
- *mean\_body\_mass\_g*: Mean individual body mass.
- *sample\_size*: Number of individuals measured.
- *reference*: Reference from which estimate of mean body mass
  were derived.

#### **raw/**

#### **avian\_nest\_monitoring/**

> `nests.shp`: Shapefile containing all nests found either
> systematically or opportunistically. See Table 2 of MetadataS1 for the
> monitoring years associated with each species.

- *nest\_id*: Unique nest identifier within a given year.
  Combining the nest\_id with the year produces a unique identifier across
  all years.
- *species*: Common species names in English, formatted in
  lowercase without dashes or apostrophes.
- *year*: Year in which the nest was found.
- *utm\_zone*: UTM zone associated with the nest
  coordinates.
- *datum*: Datum associated with the nest coordinates.

> `nests.csv`: Coordinates of all nests found systematically
> or opportunistically on Bylot Island. See Table 2 of MetadataS1 for the
> monitoring years associated with each species.

- *nest\_id*: Unique nest identifier within a given year.
  Combining the nest\_id with the year produces a unique identifier across
  all years.
- *species*: Common species names in English, formatted in
  lowercase without dashes or apostrophes.
- *year*: Year in which the nest was found.
- *lat*: Latitude of the nest location, projected in NAD27 /
  UTM zone 17N coordinate system (EPSG: 26717).
- *long*: Longitude of the nest location, projected in NAD27 /
  UTM zone 17N coordinate system (EPSG: 26717).
- *utm\_zone*: UTM zone associated with the nest
  coordinates.
- *datum*: Datum associated with the nest coordinates.

> `hawk_falcon_nests.csv`: All nests of Rough-legged hawks
> and Peregrine falcons found on Bylot Island with the corresponding zone
> of the study area. See Table 2 of MetadataS1 for the monitoring years
> associated with each species.

- *nest\_id*: Unique nest identifier within a given year.
  Combining the nest\_id with the year produces a unique identifier across
  all years.
- *species*: Common species names in English, formatted in
  lowercase without dashes or apostrophes.
- *year*: Year in which the nest was found.
- *zone*: Zone of the study area where the nest was found. The
  spatial boundaries of the study area zones are provided in
  Data\_S1/study\_area/study\_area.shp.

> `snow_goose_nests_plots.csv`: Systematic snow goose nest
> monitoring plots (see section b. Avian nest monitoring / Snow goose for
> more details).

- *Year*: Year in which the plot was sampled.
- *Habitat*: Dominant habitat in the plot (Mesic or
  Wetland).
- *plot\_number*: Sequential plot number.
- *R\_plot\_ID*: Unique plot identifier within a given year.
- *nb\_nest*: Total number of snow goose nests found in the
  plot.
- *area\_km2*: Surface area (km2) of the plot that was
  sampled.

> `summary_nest_monitoring.csv`: Summary of monitoring
> efforts for each species in the vertebrate community of Bylot Island.
> This file was used to generate Table 2 in the MetadataS1 document.

- *species*: Common species names in English, formatted in
  lowercase without dashes or apostrophes.
- *zone*: Zone of the study area where the species was
  monitored.
- *year*: Years in which the species was monitored. A dash
  indicates a continuous range (e.g., 2005–2010 means monitoring occurred
  each year from 2005 to 2010).
- *number\_years*: Total number of years in which the species
  was monitored.
- *monitoring*: Type of monitoring conducted, distinguishing
  between systematic (planned, standardized data collection) and
  opportunistic (irregular or incidental).

> `summary_nest_monitoring_long_format.csv`: Summary of
> monitoring efforts for each species in the vertebrate community of Bylot
> Island formatted in a long format.

- *species*: Species monitored. We use common species names in
  English, formatted in lowercase without dashes or apostrophes.
- *zone*: Zone of the study area where the species was
  monitored.
- *year*: Year during which the species was monitored.
- *monitored*: Indicates whether the species was monitored in
  the corresponding zone and year (“yes” if monitored, “no” if not).
- *monitoring*: Type of monitoring conducted, distinguishing
  between systematic (planned, standardized data collection) and
  opportunistic (irregular or incidental).

#### **observation\_of\_individuals/**

> `vertebrate_count_transects_2010_2023.shp`: Observations
> of individuals recorded along 500-meter vertebrate count transects
> between 2010 and 2023. See section c. Observation of Individuals /
> Vertebrate Count Transects in the MetadataS1 document for more
> details.

- *transect*: Unique transect identifier.
- *year*: Year in which the observation was made.
- *status*: Status of the individuals observed: “repro”” means
  the individuals were clearly showing breeding behavior, such as
  incubating eggs or defending nests. “non repro”” means the individuals
  were clearly not breeding, for example, seen in large groups without any
  breeding activity. “inconnu”” means it is uncertain or unknown whether
  the individuals were breeding or not.
- *nb\_ind*: Total number of individuals observed along the
  transect.
- *species*: Observed species. We use common species names in
  English, formatted in lowercase without dashes or apostrophes.
- *habitat*: Type of habitat where the transect is located,
  classified as either “mesic” or “wetland”. If any part of the transect
  overlaps with wetland, the habitat is labeled “wetland” regardless of
  the proportion; otherwise, it is labeled “mesic”.

> `vertebrate_count_transects_2010-2023.csv`: Same as the
> vertebrate\_count\_transects\_2010\_2023.shp formatted as .csv.

- *transect*: Unique transect identifier.
- *year*: Year in which the observation was made.
- *status*: Status of the individuals observed: “repro”” means
  the individuals were clearly showing breeding behavior, such as
  incubating eggs or defending nests. “non repro”” means the individuals
  were clearly not breeding, for example, seen in large groups without any
  breeding activity. “inconnu”” means it is uncertain or unknown whether
  the individuals were breeding or not.
- *nb\_ind*: Total number of individuals observed along the
  transect.
- *species*: Observed species. We use common species names in
  English, formatted in lowercase without dashes or apostrophes.
- *begin\_long*: Longitude of the location at the beginning of
  the transect, projected in NAD27 / UTM zone 17N coordinate system (EPSG:
  26717).
- *begin\_lat*: Latitude of the location at the beginning of the
  transect, projected in NAD27 / UTM zone 17N coordinate system (EPSG:
  26717).
- *end\_long*: Longitude of the location at the end of the
  transect, projected in NAD27 / UTM zone 17N coordinate system (EPSG:
  26717).
- *end\_lat*: Latitude of the location at the end of the
  transect, projected in NAD27 / UTM zone 17N coordinate system (EPSG:
  26717).

> `snow_goose_point_counts_2010_2023.shp`: Observations of
> breeding pairs of snow geese in 125 m radius point counts performed
> between 2010 and 2023. See section c. Observation of Individuals / Snow
> goose point count in the MetadataS1 document for more details.

- *year*: Year in which the observation was made.
- *point*: Unique point count identifier.
- *n\_oie\_125*: Total number of breeding snow geese pairs
  observed within a 125 m radius around the observer.
- *habitat*: Type of habitat where the point count is located,
  classified as either “mesic” or “wetland”. If any part of the point
  count overlaps with wetland, the habitat is labeled “wetland” regardless
  of the proportion; otherwise, it is labeled “mesic”.

> `incidental_observations_2007_2019.csv`: Incidental daily
> observations of vertebrates recorded between 2007 and 2019 on Bylot
> Island. See section c. Observation of Individuals / Incidental
> observations in the MetadataS1 document for more details. The original
> data, as well as any future updates, can be accessed at: https://nordicana.cen.ulaval.ca/fr/publication.php?doi=45645CE-A24D883A6676492E.

- *obs\_id*: Unique identifier assigned to each observation
  session.
- *species*: Observed species. We use common species names in
  English, formatted in lowercase without dashes or apostrophes.
- *zone*: Zone of the study area where the observation was
  made.
- *year*: Year in which the observation was made.
- *nb\_ind*: Total number of individuals observed during the
  observation session.
- *nb\_hours*: Number of hours spent on the field by the
  observer(s).
- *nb\_observers*: Number of observers in the group.

> `incidental_obs_relative_abundance_upland.csv`: Index of
> species abundance—measured as the number of individuals observed per
> hour of fieldwork—in upland and lowland habitats, based on incidental
> observations. Observations made during hawk nest visits were used as a
> proxy for upland habitat observations. All other observations were
> considered as lowland.

- *species*: Species observed. We use common species names in
  English, formatted in lowercase without dashes or apostrophes.
- *nb\_ind\_hour\_upland*: Number of individuals observed per hour
  when performing fieldwork in upland habitat.
- *nb\_ind\_hour\_lowland*: Number of individuals observed per
  hour when performing fieldwork in lowland habitat.
- *ratio\_difference*: Ratio between nb\_ind\_hour\_upland and
  nb\_ind\_hour\_lowland.

> `relative_abundance_Gauthier_et_al_2024.csv`: Index of
> species abundance, calculated as the number of individuals observed per
> 100 hours of fieldwork, based on incidental observations and reported in
> Gauthier et al. 2024b. The presented indices were used to compare the
> relative abundance of species with those for which absolute abundance
> was estimated (i.e., reference species).

- *species*: Common species names in English, formatted in
  lowercase without dashes or apostrophes.
- *index\_sp*: Number of individuals of the corresponding
  “species” observed per 100 hours of fieldwork.
- *ref\_species*: Reference species (i.e. a species for which
  absolute abundance has been estimated).
- *index\_ref\_sp*: Number of individuals of the corresponding
  “ref\_species” observed per 100 hours of fieldwork.

> `american_golden_plover_distance.csv`: Perpendicular
> distance (in meters) measured between the transect and American
> golden-plover individuals detected during vertebrate count
> transects.

- *distance*: Perpendicular distance (in meters) from the
  transect to the detected individuals.

> `ermine_index.csv`: Annual index of American ermine
> abundance on Bylot Island, based on local testimonials reported in
> Bolduc et al. (2023; https://doi.org/10.1016/j.baae.2022.11.005).

- *Year*: Year in which the American ermine abundance index was
  estimated.
- *Ermine*: Index of American ermine abundance based on local
  testimonials. For details on data collection and usage, refer to Bolduc
  et al. (2023).

#### **capture\_of\_individuals/**

> `lemming_density_1993-2019.csv`: Estimates of individual
> density (individuals/ha) for Nearctic brown and collared lemmings based
> on snap-trapping conducted in mesic and wetland grids of the
> Qarlikturvik Valley in late July from 1993 to 2019. See Section
> d. Capture of Individuals / Lemming Trapping for more details.

- *Year*: Year in which the density was estimated.
- *Brown Wet habitat*: Estimated density of Nearctic brown
  lemmings (individuals per hectare) within the trapping grid located in
  wetland habitat.
- *Brown Mesic habitat*: Estimated density of Nearctic brown
  lemmings (individuals per hectare) within the trapping grid located in
  mesic habitat.
- *Coll Wet habitat*: Estimated density of Nearctic collared
  lemmings (individuals per hectare) within the trapping grid located in
  wetland habitat.
- *Coll Mesic habitat*: Estimated density of Nearctic collared
  lemmings (individuals per hectare) within the trapping grid located in
  mesic habitat.
- *Both Wet habitat*: Estimated density of both Nearctic brown
  and collared lemmings combined (individuals per hectare) within the
  trapping grid located in wetland habitat.
- *Both Mesic habitat*: Estimated density of both Nearctic
  brown and collared lemmings combined (individuals per hectare) within
  the trapping grid located in mesic habitat.

> `lemming_density_2004-2022.csv`: Estimates of individual
> density (individuals/ha) for Nearctic brown and collared lemmings
> derived from live-trapping conducted between 2004 and 2022. See Section
> d. Capture of Individuals / Lemming Trapping for more details.

- *site*: Zone of the study area where trapping was conducted.
  Note that Goose camp here refer here to the trapping grids located in
  the Qarlikturvik valley.
- *species*: Lemming species, either “brown” or “collared” for
  Nearctic brown and Nearctic collared lemming respectively.
- *grid*: Unique identifier assigned of each trapping
  grid.
- *habitat*: Type of habitat where the trapping grid was
  located.
- *year*: Year during which trapping was conducted.
- *period*: Period during which trapping was conducted (“P1”=
  mid-June; “P2”= mid-July and “P3”= mid-August).
- *density*: Estimated density of individuals (individuals per
  hectare).
- *se*: Standard error associated with the estimate.

> `data_HomeRange.csv`: Predicted Arctic fox home range
> sizes inside and outside the goose colony during years of low and high
> lemming density. The dataset is available at https://doi.org/10.5061/dryad.f1vhhmh30. For details and
> data usage, see Dulude-de Broin et al. (2023; https://doi.org/10.1111/1365-2656.14017) and section a.
> Field/laboratory / Arctic fox.

#### **body\_mass/**

> `body_mass_measurements_bylot.csv`: Body mass (in grams)
> of individuals measured on Bylot Island. The dataset also includes the
> average body mass of Cackling Geese captured on Baffin Island. For
> details on Cackling Goose measurements, see Neufeld (2021; http://hdl.handle.net/1993/35933).

- *species*: Common species names in English, formatted in
  lowercase without dashes or apostrophes.
- *age*: Age class of the individuals. “adult” indicates
  breeding individuals, while “NA” is used when the distinction between
  adults and juveniles was not available.
- *site*: Location where the measurement was taken. “bylot”
  refers to the study area on the southern plain of Bylot Island, while
  “baffin” refers to data for Cackling Goose from Baffin Island (see
  Neufeld, 2021).
- *year*: Year in which the measurement was taken.
- *body\_mass\_g*: Body mass of the individual, measured in
  grams.
- *reference*: Source or reference associated with the
  measurement.

#### **metadata/**

> `summary_methods.csv`: Metadata used to generate Table 4
> in the MetadataS1 document.

#### **study\_area/**

> `study_area.shp`: Spatial boundaries of the different
> zones of the study area on the southern plain of Bylot Island, Nunavut,
> Canada.

- *zone*: Zone of the study area.
- *year*: Year in which the snow goose colony area was
  delineated. “NA” for all zones except the “goose colony.”
- *area\_km2*: Surface area of the zone, in square
  kilometers.
- *prop\_wet*: Proportion of wetland habitat within the
  zone.
- *prop\_mesic*: Proportion of mesic habitat within the
  zone.
- *prop\_up*: Proportion of upland habitat within the zone.

> `lakes.shp`: Spatial boundaries of lakes and ponds within
> the study area on the southern plain of Bylot Island, Nunavut, Canada
> (see Corbeil-Robitaille et al., 2024).

> `rivers.shp`: Spatial boundaries of lakes and ponds within
> the study area on the southern plain of Bylot Island, Nunavut,
> Canada.

> `wetlands.shp`: Spatial boundaries of wetlands within the
> study area on the southern plain of Bylot Island, Nunavut, Canada
> (Louis-Pierre Ouellet, unpublished data).

> `proportion_goose_colony_zone.csv`: Annual proportion of
> each zone of the study area overlapping with the delineated snow goose
> colony.

- *zone*: Zone of the study area.
- *year*: Year in which the snow goose colony area was
  delineated.
- *prop\_overlap\_colony*: Proportion of the zone that overlaps
  with the goose colony boundary.

> `snow_goose_systematic_wetland_plot.shp`: Spatial
> delineation of the primary wetland plot used for systematic monitoring
> of snow goose nests. All nests located within this plot are monitored
> annually.

#### **script/**

#### **1\_data\_cleaning/**

> `1.1_clean_year_monitoring_metadata.R`: R script used to
> convert the compact summary table of species monitoring efforts into a
> long-format suitable for manipulation in R.

> `1.2_extract_species_body_mass.R`: R script to extract the
> mean individual body mass (in grams) for each species in the vertebrate
> community of Bylot Island.

> `1.3_detection function_american_golden_plover.R`: R
> script used to identify the parameter combination that yields the lowest
> AIC values in the distance sampling model for American
> golden-plover.

#### **2\_extract\_species\_density\_abundance/**

> `2.1_extract_species_density_monitoring.R`: Extract annual
> density of individuals measured in the different zones of the study area
> (see BYLOT-species\_density\_monitoring.csv).

> `2.2_extract_species_abundance.R`: Estimate the annual or
> mean abundance of all vertebrate species in the community at the scale
> of the study area (389 km2; see BYLOT-species\_abundance.csv).

> `2.3_extract_community_composition.R`: Extract the annual
> or mean abundance of all vertebrate species in the Bylot Island
> community using the method with the highest quality rating, when
> applicable (see BYLOT-community\_composition.csv).

#### **species/**

> `american_ermine.R`: Script used to estimate American
> ermine abundance across the entire study area.

> `american_golden_plover.R`: Script used to estimate
> American golden-plover abundance across the entire study area.

> `arctic_fox.R`: Script used to estimate Arctic fox
> abundance across the entire study area.

> `arctic_hare.R`: Script used to estimate Arctic hare
> abundance across the entire study area.

> `bairds_sandpiper.R`: Script used to estimate Baird’s
> sandpiper abundance across the entire study area.

> `black_bellied_plover.R`: Script used to estimate
> Black-bellied plover abundance across the entire study area.

> `cackling_goose.R`: Script used to estimate Cackling goose
> abundance across the entire study area.

> `common_raven.R`: Script used to estimate Common raven
> abundance across the entire study area.

> `common_ringed_plover.R`: Script used to estimate
> Common-ringed plover abundance across the entire study area.

> `ducks.R`: Script used to estimate the abundance of King
> eider and Long-tailed duck across the entire study area.

> `glaucous_gull.R`: Script used to estimate Glaucous gull
> abundance across the entire study area.

> `lapland_longspur.R`: Script used to estimate Lapland
> longspur abundance across the entire study area.

> `lemmings.R`: Script used to estimate the abundance of
> Nearctic brown lemming and Nearctic collared lemming across the entire
> study area.

> `long_tailed_jaeger.R`: Script used to estimate
> Long-tailed jaeger abundance across the entire study area.

> `loons.R`: Script used to estimate the abundance of
> Pacific loon and Red-throated loon across the entire study area.

> `other_passerine.R`: Script used to estimate the abundance
> of Horned lark, American pipit and Snow bunting across the entire study
> area.

> `other_sandpiper.R`: Script used to estimate the abundance
> of Ruddy turnstone, Red knot, Pectoral sandpiper, White-rumped
> sandpiper, Buff-breasted sandpiper and Red phalarope across the entire
> study area.

> `parasitic_jaeger.R`: Script used to estimate Parasitic
> jaeger abundance across the entire study area.

> `peregrine_falcon.R`: Script used to estimate Peregrine
> falcon abundance across the entire study area.

> `rock_ptarmigan.R`: Script used to estimate Rock ptarmigan
> abundance across the entire study area.

> `rough_legged_hawk.R`: Script used to estimate
> Rough-legged hawk abundance across the entire study area.

> `sandhill_crane.R`: Script used to estimate Sandhill crane
> abundance across the entire study area.

> `snow_goose.R`: Script used to estimate Snow goose
> abundance across the entire study area.

> `snowy_owl.R`: Script used to estimate Snowy owl abundance
> across the entire study area.

> `tundra_swan.R`: Script used to estimate Tundra swan
> abundance across the entire study area.

#### **3\_tables\_figures/**

> `3.1_table_species_name_strategy.R`: Code to generate
> Table 1 in the MetadataS1 document.

> `3.2_table_species_year_monitoring.R`: Code to generate
> Table 2 in the MetadataS1 document.

> `3.3_table_species_relative_abundance_upland.R`: Code to
> generate Table 3 in the MetadataS1 document.

> `3.4_table_summary_methods_and_abundance.R`: Code to
> generate Table 4 in the MetadataS1 document.

> `3.5_figures_species_temporal_series.R`: Code to generate
> Figure 11 in the MetadataS1 document.

> `3.6_maps.R`: Code to generate Figure 3, 4, 5, 6 in the
> MetadataS1 document.

> `3.7_table_variables_definition.R`: Code to generate Table
> 6 in the MetadataS1 document.

> `3.8_table_variables_format.R`: Code to generate Table 7
> in the MetadataS1 document.

> `3.9_table_expert_based_uncertainty.R`: Code to generate
> Table 5 in the MetadataS1 document.

> `3.10_figure_time_line.R`: Code to generate Table 5 in the
> MetadataS1 document.

#### **functions/**

> `FUNS.R`: Code loading all local functions found in the
> “standalone\_functions” and “nested\_functions”.

#### **standalone\_functions/**

> `get_nest_density.R`: R function to extract the density of
> nests and individuals for a given list of zones and years.

> `get_nest_density_missing_coordinates.R`: R function to
> calculate the density of nests and individuals for a given list of zones
> and years when exact coordinates of nests are missing.

> `number_ind_transects.R`: R function to calculate the mean
> number of individual observed per transect for a given list of zones and
> years.

> `extract_density_distance_sampling.R`: R function to
> estimate the abundance of individuals for a given list of zones and
> years based on distance sampling.

#### **nested\_functions/**

> `extend_temporal_series_from_single_zone_nest.R`: R
> function used to extend a temporal abundance series by scaling nest
> density measured in a single zone to the entire study area, using the
> relationship between densities at the two spatial scales.

> `extend_temporal_series_from_single_zone_missing_coordinates.R`:
> R function used to extend a temporal abundance series by scaling nest
> density measured in a single zone to the entire study area, using the
> relationship between densities at the two spatial scales; when exact
> nests coordinates are missing (Rough-legged hawks and Peregrine
> falcons).

> `spatial_extrapolation_species_density.R`: R function used
> to extrapolate local nest density to the entire study area based on
> observations collected along transects across different zones.

> `estimate_density_from_reference_species.R`: R function
> used to estimate the absolute abundance of a given species by applying
> the ratio of its relative abundance to that of a reference species with
> known absolute abundance.

### Sharing/access information

The dataset is also available as supplementary information in the
related data paper (Moisan et al., 2025, Ecology).

### Version history (Changelog)

#### *Version 1 (19-11-2024)*

- The data set (`Data_S1/`) includes originally only the
  following files:   
   `BYLOT-species_taxonomy.csv`,  
  `BYLOT-species_abundance.csv`,  
  `BYLOT-species_body_mass.csv` and  
  `BYLOT-interannual_variation_nest_density.csv`.

#### *Version 2 (23-06-2025)*

- The folders `raw/`, `study_area/` and
  `metadata/` were added to `Data_S1/`. These
  folders and files were previously archived on Zenodo.
- The following objective was added:
- `BYLOT-interannual_variation_nest_density.csv` was
  replaced by `BYLOT-species_density_monitoring.csv`, which now
  provides raw measurements of species density (instead of only mean and
  standard deviation).
- `BYLOT-community_composition.csv` was added, presenting
  full tabular estimates of annual or mean species abundance at the
  landscape scale (389 km²).
- A new column `spatial_extrapolation`, indicating whether
  estimates of abundance were spatially extrapolated, was added to
  `BYLOT-species_abundance.csv`.
- The method used to estimate cackling goose abundance at the
  landscape scale between 2004 and 2016 was updated; corresponding values
  were modified in `BYLOT-species_abundance.csv`.
- Null lemming density values were set to 0.025 individuals/ha when
  live trapping was conducted (after 2004), representing half of the
  lowest lemming density detectable with our sampling scheme.
- The average body mass of cackling goose measured on Baffin Island
  was added to `BYLOT-species_body_mass.csv`.
- Timelines representing the time series of each species in the
  dataset (Figure 2), and a map showing the distribution of habitat types
  across the study area (Figure 3), were added to
  `MetadataS1.pdf`.
- Minor clarifications and edits were made throughout
  `MetadataS1.pdf`.

#### *Version 3 (11-08-2025)*

- The column species\_code was removed from
  `BYLOT-species_taxonomy.csv`.
- The manuscript has been accepted for publication; minor details were
  corrected following final revision by the subject-matter editor, the
  authors and the editorial staff.

#### *Version 4 (12-08-2025)*

- The hyperlink to the complete R project, which previously redirected
  to version 1 of the repository (https://zenodo.org/records/13993827), was updated to
  point to the general software repository containing the up-to-date
  version (https://doi.org/10.5281/zenodo.13993826).

### Citation

Please use the following citation when referencing this data set:

Moisan, L., Bideault, A., Gauthier, G., Duchesne, É., Fauteux, D.,
Berteaux, D., Legagneux, P., Cadieux, M.-C. and Bêty, J. (2025).
Long-term abundance time-series of the High Arctic terrestrial
vertebrate community of Bylot Island, Nunavut. Ecology.

### Code/Software

*Operating system*: Data preparation was performed on
x86\_64-pc-linux-gnu (64-bit) with Ubuntu 22.04.3 LTS.

*Program*: R version 4.4.1 (2024-06-14)

*Packages*:

### Contact persons

*Overall project*: Joël Bêty (joel\_bety@uqar.ca)

*Data set and code*: Louis Moisan (louis.moisan.bio@gmail.com)

### References

Bates, D., Mächler, M., Bolker, B., and Walker, S. (2015). Fitting
linear mixed-effects models using lme4. Journal of Statistical Software,
67(1):1–48.

Bolduc, D., Fauteux, D., Gagnon, C. A., Gauthier, G., Bêty, J., and
Legagneux, P. (2023). Testimonials to reconstruct past abundances of
wildlife populations. Basic and Applied Ecology, 68:23–34.

Corbeil-Robitaille, M.-Z., Duchesne, É., Fortier, D., Kinnard, C.,
and Bêty, J. (2024). Linking geomorphological processes and wildlife
microhabitat selection: nesting birds select refuges generated by
permafrost degradation in the arctic. Biogeosciences,
21(14):3401–3423.

Dahl, D. B., Scott, D., Roosen, C., Magnusson, A., and Swinton, J.
(2019). xtable: Export Tables to LaTeX or HTML. R package version
1.8-4.

Dulude-de Broin, F., Clermont, J., Beardsell, A., Ouellet, L.-P.,
Legagneux, P., Bêty, J., and Berteaux, D. (2023). Predator home range
size mediates indirect interactions between prey species in an arctic
vertebrate community. Journal of Animal Ecology, 92(12):2373–2385.

Dunnington, D. (2023). ggspatial: Spatial Data Framework for ggplot2.
R package version 1.1.9.

Gauthier, G., Berteaux, D., Bêty, J., Tarroux, A., Therrien, J.-F.,
McKinnon, L., Legagneux, P., and Cadieux, M.-C. (2011). The tundra food
web of bylot island in a changing climate and the role of exchanges
between ecosystems. Ecoscience, 18(3):223–235.

Gauthier, G., Cadieux, M.-C., Berteaux, D., Bêty, J., Fauteux, D.,
Legagneux, P., Lévesque, E., and Gagnon, C.-A. (2024b). Long-term study
of the tundra food web at a hotspot of arctic biodiversity, the bylot
island field station. Arctic Science, 10(1):108–124.

Gill, F., Donsker, D., and Rasmussen, P. (2024). Ioc world bird list
(v14. 2).

Kahle, D. and Wickham, H. (2013). ggmap: Spatial visualization with
ggplot2. The R Journal, 5(1):144–161.

Mazerolle, M. J. (2023). AICcmodavg: Model selection and multimodel
inference based on (Q)AIC(c). R package version 2.3.3.

Miller, D. L., Rexstad, E., Thomas, L., Marshall, L., and Laake, J.
L. (2019). Distance sampling in R. Journal of Statistical Software,
89(1):1–28.

Moisan, L., Gravel, D., Legagneux, P., Gauthier, G., Léandri-Breton,
D.-J., Somveille, M., Therrien, J.-F., Lamarre, J.-F., and Bêty, J.
(2023). Scaling migrations to communities: An empirical case of
migration network in the arctic. Frontiers in Ecology and Evolution,
10:1077260.

Neufeld, L. (2021). Comparing migration ecology among geographically
distinct populations of Canada Geese (Branta canadensis) and Cackling
Geese (Branta hutchinsii). Master’s thesis, University of Manitoba.

Pebesma, E. J. et al. (2018). Simple features for r: standardized
support for spatial vector data. The R Journal, 10(1):439–446.

Upham, N., Burgin, C., Widness, J., Liphardt, S., Parker, C., Becker,
M., Rochon, I., Huckaby, D., and Zijlstra, J. (2024). Mammal diversity
database.

Wickham, H. (2016). ggplot2: Elegant Graphics for Data Analysis.
Springer-Verlag New, ISBN 978-3-319-24277-4. York.

Wickham, H., François, R., Henry, L., Müller, K., and Vaughan, D.
(2023a). dplyr: A Grammar of Data Manipulation. R package version
1.1.4.

Wickham, H., Pedersen, T. L., and Seidel, D. (2023b). scales: Scale
Functions for Visualization. R package version 1.3.0.

Wickham, H. (2023). stringr: Simple, Consistent Wrappers for Common
String Operations. R package version 1.5.1.

Wickham, H., Vaughan, D., and Girlich, M. (2024). tidyr: Tidy Messy
Data. R package version 1.3.1.
