# Supplementary material for: Long‐term abundance time‐series of the High Arctic terrestrial vertebrate community of Bylot Island, Nunavut
Source: Ecology. 2025 Oct 7;106(10):e70223. doi: 10.1002/ecy.70223 (PMC12501677; doi:10.1002/ecy.70223)
Supplement: Supplementary file 1 — Data S1: [file ECY-106-e70223-s001.zip › MetadataS1.pdf]

Data Paper published in *Ecology*, an Ecological Society of Americal journal

Metadata S1

Long-term abundance time-series of the High Arctic terrestrial vertebrate community of Bylot Island, Nunavut

Louis Moisan, Azenor Bideault, Gilles Gauthier, Éliane Duchesne, Dominique Fauteux, Dominique Berteaux, Pierre Legagneux, Marie-Christine Cadieux, and Joël Bêty

Open Research: The complete data set is available in Data S1 as Supporting Information. The complete data set, including raw data, is also archived in Dryad at https://doi.org/10.5061/dryad.44j0zpcnt. Comprehensive documentation on the study context, objectives, methods, and metadata is archived in Zenodo at https://doi.org/10.5281/zenodo.16794620. The code and the complete R project used to estimate species abundance are archived in Zenodo at https://doi.org/10.5281/zenodo.16794619. Moreover, long-term climatic data within the study area are available in the NordicanaD repository at https://doi.org/10.5885/45039SL-EE76C1BDAADC4890. Finally, raw monitoring data for the following key species of the food web are also available at the NordicanaD data repository (https://nordicana.cen.ulaval.ca/en/list-of-publications.php) and are periodically updated as the field studies continue on Bylot Island: Lemming monitoring on Bylot Island; Monitoring of Greater Snow Goose reproduction on Bylot Island; Monitoring of Lapland longspur reproduction on Bylot Island; Monitoring of shorebirds reproduction on Bylot Island; Monitoring of arctic and red fox reproduction on Bylot Island; Monitoring of avian predator reproduction on Bylot Island; Relative abundance of tundra bird and mammal species encountered daily on Bylot Island.

Introduction

The composition of ecological communities, defined as the abundance of each species within a given community, is fundamental for understanding patterns and processes in community ecology. Variations in community composition can help to detect spatial patterns linked to environmental variations (Kemp et al., 1990), assess temporal trends of different groups of species after disturbances (Philippi et al., 1998; Magurran, 2007), and understand food web structures (Cohen et al., 2003). Additionally, species composition and their relative abundance are essential for modeling the dynamics of ecological communities. Dynamic community modelling allows addressing important issues and questions in ecology, such as: determining the relative strength of top-down versus bottom-up forces in communities (Krebs et al., 2003; Legagneux et al., 2014), assessing the ecological resilience of communities under climate change (Griffith et al., 2019) and evaluating the cascading effects of invasive species in food web (David et al., 2017; Goto et al., 2020). Dynamic community modelling can also be applied to address practical challenges, including fishery management (Plagányi, 2007) and the planning of protected areas (Okey et al., 2004; Dahood et al., 2020).

Modeling food webs requires adjusting trophic flows based on the functional or numeric responses of species, which necessitates time series data on the abundance of all species within a community. However, determining the abundance of all species within a community is rarely achievable. Consequently, empirical community models often reduce taxonomic resolution by grouping species into large functional or taxonomic categories. Additionally, food webs consist of species with varying body sizes depending on their trophic level, with top-level species often being highly mobile and having large home ranges (McCann et al., 2005). Therefore, community models must use landscape-wide estimates of species abundance to accurately represent trophic fluxes. Due to these constraints, empirical datasets with high taxonomic resolution that cover entire communities at broad spatial and temporal scales are rare and often include incomplete or rough estimates.

The composition of ecological communities is influenced by various factors acting at different temporal and spatial scales, leading to noisy data and emphasizing the need for long-term data sets (Magurran et al., 2010; Lindenmayer et al., 2012). Species abundances are influenced by stochastic effects (Hubbell, 2001), environmental changes (e.g., climate warming), and species interactions, contributing to data variability. For instance, the composition of a community could be driven simultaneously by intra-annual seasonal variations, multi-year cyclic variations (e.g., El Niño) and slow but directional long-term variations in the environment (Brown and Heske, 1990; Snyder and Tartowski, 2006). Therefore, long-term data series are required to untangle the relative effects of diverse

abiotic and biotic factors on community composition (Magurran et al., 2010; Lindenmayer et al., 2012).

Arctic environments are highly valuable systems for studying community structure and dynamics due to their relatively low species richness (Payer et al., 2013; Legagneux et al., 2014). However, logistical challenges in the Arctic limit the number or length of biodiversity monitoring programs. Hence, the small number of Arctic communities with long-term monitoring serve as highly valuable sites for holistic and empirical community studies. Datasets on terrestrial communities are notably scarce, and this scarcity extends to Arctic communities as well (Ims et al., 2013).

The south plain of Bylot Island in the Canadian High Arctic (**Figure 1**) hosts one of the longest and most intensive vertebrate monitoring programs in the High-Arctic (Gauthier et al., 2024b). Monitoring on Bylot Island began in 1989 with a focus on the snow goose and it gradually expanded to other species over time. The program currently encompasses all vertebrate species in the community except for rare and occasional species (Gauthier et al., 2011; Legagneux et al., 2012), with continuous monitoring spanning more than a decade for several species (Gauthier et al., 2024b). Monitoring is also conducted at multiple spatial scales, including intensive and systematic observations conducted across a landscape spanning approximately 400 km^2. This approach enables local density measurements to be scaled to the landscape level by incorporating relative abundance across the landscape.

Previous work based on the tundra community of Bylot Island has already produced several influential papers (Gauthier et al., 2011, 2013; Legagneux et al., 2012, 2014; Hutchison et al., 2020; Duchesne et al., 2021; Gauthier et al., 2024a). These studies showed that tundra communities may experience stronger top-down regulation than bottom-up regulation (Legagneux et al., 2012, 2014). They also revealed a heterogeneous response of trophic levels to climate warming (Gauthier et al., 2013) and highlighted the effects of indirect trophic interactions on the occurrence of species across the landscape (Duchesne et al., 2021). However, those earlier papers were built on data from relatively short time series, they were not always scaled at the landscape level, and some species or functional groups were lacking abundance estimates.

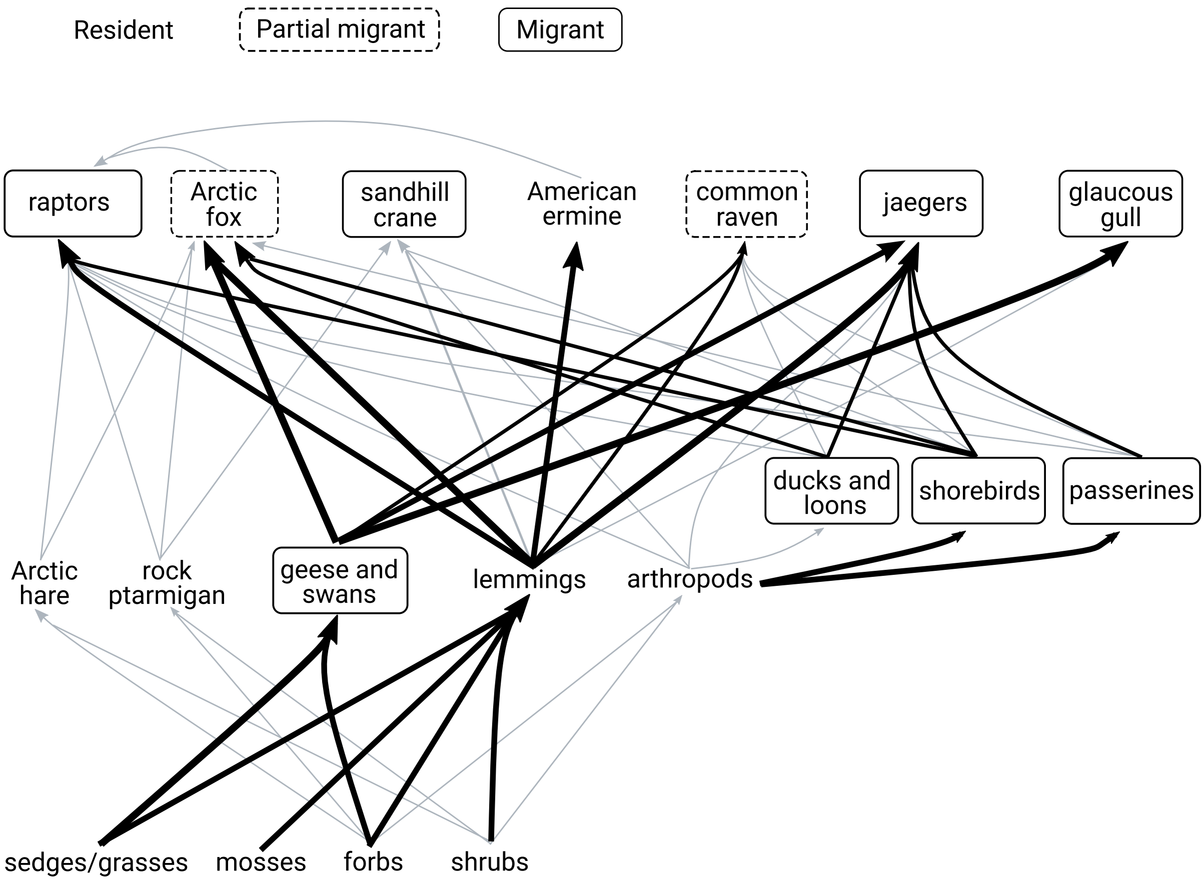

Figure 1: Synthetic vertebrate food web of the south plain of Bylot Island. The figure was adapted from Moisan et al. (2023) and Gauthier et al. (2011).

Objectives

With over a decade of additional community-wide monitoring compared to earlier studies, our goals are to i) provide long-term time series of annual vertebrate density measured at various spatial scales and for the broadest possible range of species and years, to allow the assessment of interannual variability and trends in species density; and ii) upscale annual vertebrate abundance or sometimes long-term averages to the landscape scale (400 km^2) to allow food web modelling (**Figure 2**). We focus on the breeding season (May to August) here because it is the most appropriate period to conduct surveys for most species, especially migratory birds. Although we recognize that ecological dynamics during the non-breeding period, for both resident (Hutchison et al., 2020) and migratory species (Moisan et al., 2023), can influence the food web during the breeding season, it was not possible to cover this period in the present study. The abundance estimates include both breeding and non-breeding individuals that remain in the study area for a significant period of time (e.g., territorial breeding or non-breeding foxes), and excludes non-breeding individuals that stop only briefly during migration (e.g., shorebirds using Bylot Island for a short stopover) or leave the area shortly after arrival without attempting to breed in

some years (e.g. long-tailed jaegers). We focus on adults, except for lemmings for which we have not distinguished between juveniles and adults. Additionally, we aim to provide the average body mass for each species in the community, enabling the conversion of abundances into biomasses.

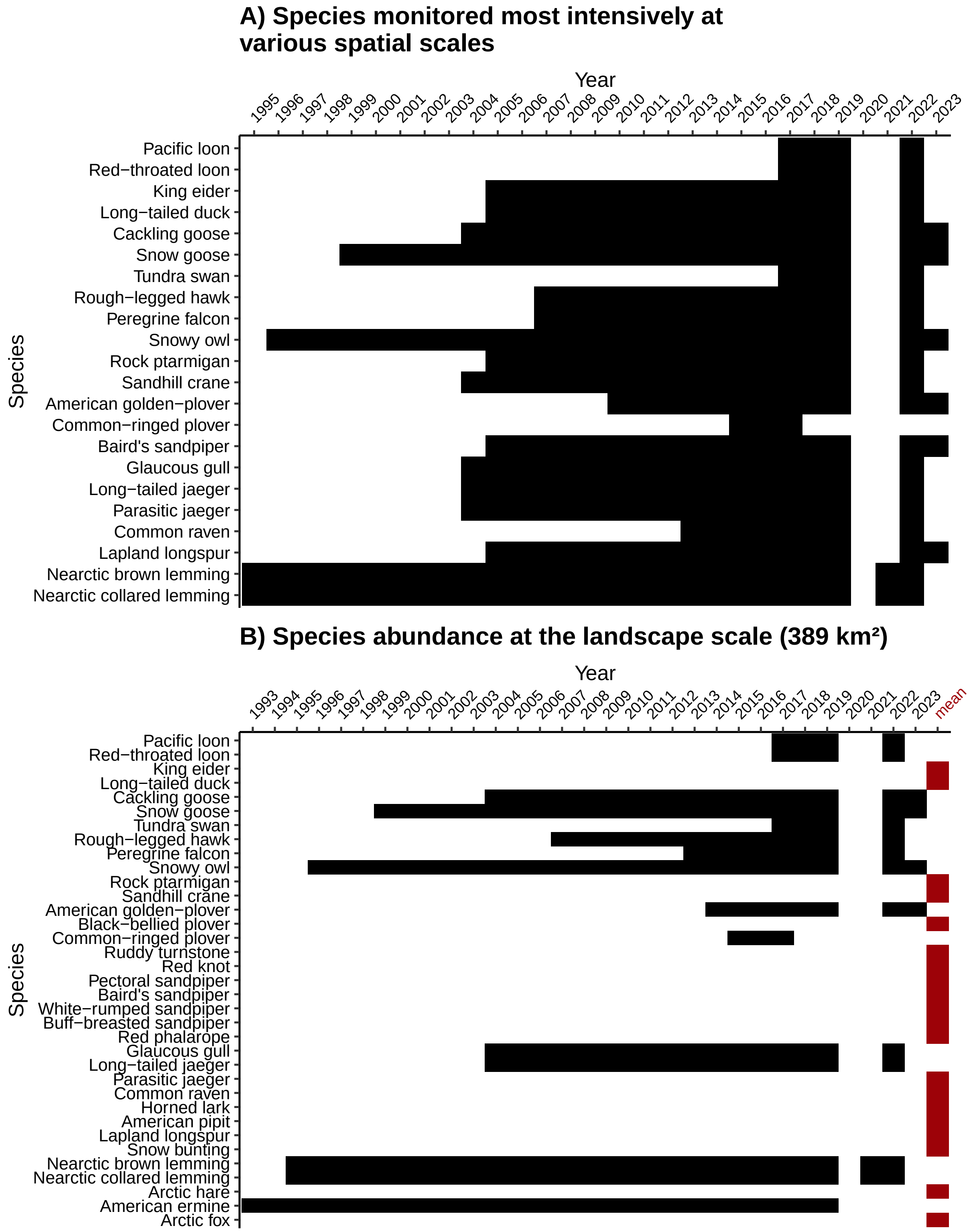

Figure 2: A) Time series showing the longest available density records for 22 vertebrate species monitored in the community of Bylot Island. The spatial scale (i.e., zones of the study area) at which density was measured varies among species (see BYLOT-species_density_monitoring.csv). B) Time series of annual species abundance (black) or mean species abundance (red) at the landscape level (389 km^2) for all 35 vertebrate species in the community (see BYLOT-community_composition.csv).

Class I. Data Set Descriptors

A. Data set identity

Long-term abundance time-series of the High Arctic terrestrial vertebrate community of Bylot Island, Nunavut

B. Data set identification codes

BYLOT-species_taxonomy.csv
BYLOT-species_density_monitoring.csv
BYLOT-species_abundance.csv
BYLOT-community_composition.csv
BYLOT-species_body_mass.csv

C. Data set description

1. Originators

Gilles Gauthier, Centre d'Études Nordiques, Département de Biologie, Université Laval, Québec, QC, Canada
Joël Bêty, Chaire de Recherche du Canada en Biodiversité Nordique, Centre d'Études Nordiques, Centre de la Science de la Biodiversité du Québec, Département de Biologie, Chimie et Géographie, Université du Québec à Rimouski, Rimouski, QC, Canada
Pierre Legagneux, Chaire de Recherche Sentinelle Nord sur l'Impact des Migrations Animales sur les Écosystèmes Nordiques, Centre d'Études Nordiques, Centre de la Science de la Biodiversité du Québec, Centre d'Études Biologiques de Chizé (CEBC-CNRS) Département de Biologie, Université Laval, Québec, QC, Canada
Dominique Berteaux, Chaire de Recherche du Canada en Biodiversité Nordique, Centre d'Études Nordiques, Centre de la Science de la Biodiversité du Québec, Département de Biologie, Chimie et Géographie, Université du Québec à Rimouski, Rimouski, QC, Canada

2. Abstract

Arctic ecosystems present unique opportunities for community-wide monitoring, in part due to their relatively low species richness. However, conducting research in these remote environments poses significant logistical challenges, resulting in long-term monitoring being exceedingly rare. Here, we focus on the long-term, intensive ecological monitoring efforts conducted on the south plain of Bylot Island (~400 km^2, Nunavut, Canada), which has generated a remarkable dataset spanning up to 30 years, a rarity in tundra ecosystems. Our goals are to i) provide long-term time series of annual vertebrate density measured at various spatial scales and for the broadest possible range of species and years, to allow the assessment of interannual variability and trends in species density; and ii) upscale annual vertebrate abundance or sometimes long-term averages to the landscape scale (400 km^2) to allow food web modelling. Monitoring data includes intensive capture-mark-recapture density estimates of lemmings on trapping grids, systematic or opportunistic nest monitoring conducted across the entire study area or within specific plots for all bird species, transects of vertebrate counts distributed throughout the study area, daily incidental observations of vertebrates and satellite tracking of foxes. We standardized data obtained with different field methods to provide a readily usable data set for community ecologists. Long-term time series of vertebrate densities span 3 to 27 years, with a median of 16.5 years for 22 species. We estimated landscape-scale abundance for all 35 species of the community based on annual time series for 15 of them and average abundance for the remaining 20 species. Furthermore, we provide body mass data for each species, based on empirical onsite measurements for 18 species and from the literature for the remaining species. Body mass is essential to convert species abundance into biomass for studies of trophic fluxes and ecosystem processes. Daily climatic data recorded since 1992 from weather stations within the study area are also available and complement the vertebrate dataset. The ecological data presented offer a rare opportunity for holistic empirical studies of community structure and dynamics. Considering that the study site is a pristine and protected area that has experienced minimal direct anthropogenic impact, it also provides an ideal baseline for investigating the impacts of global changes on high-latitude terrestrial ecosystems. There are no copyright restrictions on the data or code, and this data paper should be cited when these items are reused.

D. Key words/phrases

Arctic tundra, Biodiversity monitoring, Bylot Island, Canadian Arctic, Community composition, Community structure, Food web, Long-term monitoring, Species abundance, Species biomass, Species body mass

Class II. Research origin descriptors

A. Overall project description

1. Identity

Structure and functioning of Arctic terrestrial ecosystems

2. Originators

Gilles Gauthier, Centre d'études nordiques, Département de Biologie, Université Laval, Québec, QC, Canada
Joël Bêty, Chaire de Recherche du Canada en Biodiversité Nordique, Centre d'Études Nordiques, Centre de la science de la biodiversité du Québec, Département de biologie, chimie et géographie, Université du Québec à Rimouski, Rimouski, QC, Canada
Pierre Legagneux, Chaire de Recherche Sentinelle Nord sur l'impact des migrations animales sur les écosystèmes nordiques, Centre d'Études Nordiques, Centre de la science de la biodiversité du Québec, Centre d'Études Biologiques de Chizé (CEBC-CNRS) Département de Biologie, Université Laval, Québec, QC, Canada
Dominique Berteaux, Chaire de Recherche du Canada en Biodiversité Nordique, Centre d'Études Nordiques, Centre de la Science de la Biodiversité du Québec, Département de Biologie, Chimie et Géographie, Université du Québec à Rimouski, Rimouski, QC, Canada

3. Period of study

1989 - continuing

4. Objectives

i) Develop models for the Arctic tundra food web that incorporate seasonality, migratory connectivity and interactions with marine and southern ecosystems.
ii) Predict the effects of global changes on Arctic terrestrial food webs.

5. Abstract

Arctic terrestrial communities, characterized by relatively low species richness, offer unique opportunities for studying ecological patterns and community dynamics in simplified systems. Despite their relative simplicity, these ecosystems feature complex species interac-

tions, extreme seasonal environmental changes, and a significant proportion of migratory species, making it difficult to identify the key factors shaping their structure and functioning. As global environmental changes accelerate, it is essential to understand the interacting processes driving these communities to eventually predict future impacts on Arctic ecosystems. Our research combines long-term biodiversity monitoring, a community-wide approach, and food web modeling to address these challenges.

6. Sources of funding

Natural Sciences and Engineering Research Council of Canada, Fonds Québécois de Recherche Nature et Technologies, Centre d'études nordiques, Natural Resources Canada (Polar Continental Shelf Program), Network of Centers of Excellence Canada (ArcticNet), Canada First Research Excellence Fund (Sentinel North Program), Polar Knowledge Canada, Environment and Climate Change Canada, Canada Foundation for Innovation, Parks Canada Agency, International Polar Year program of the Government of Canada, Crown–Indigenous Relations and Northern Affairs Canada (Northern Contaminant Program), Ducks Unlimited Canada, Kenneth M. Molson Foundation (Kenneth M. Molson Foundation's donation for wildlife research, conservation, and habitat), Garfield Weston Foundation, First Air-Canadian North, Nunavut Wildlife Management Board, Université Laval, Université du Québec à Rimouski

B. Specific subproject description

1. Site description

a. Site type

The study area (389 km^2) represents a relatively productive tundra ecosystem compared to other sites at similar latitudes in the eastern Canadian High Arctic (Gauthier et al., 2024b). An important biological characteristic of the area is the presence of a large snow goose (scientific names of most vertebrate species can be found in **Table 1**) colony of around 25 000 breeding pairs (Reed et al., 2002) spanning approximately 70 km^2. The vertebrate community within the study area comprises 30 bird species, with 29 of them being migratory or partially migratory, along with 5 mammal species (**Table 1**; Moisan et al. 2023; Gauthier et al. 2024b). The study area experiences significant temporal fluctuations in the population of small mammals (lemmings), which in turn impact the occurrence and abundance of their avian and mammalian predators such as snowy owls, rough-legged hawks, long-tailed jaegers and ermines (Therrien et al., 2014; Duchesne et al., 2021; Bolduc et al., 2025). We exclude occasional visitors, namely: i) species lacking confirmed breeding occurrences on the study site, ii) species observed solely within a single year, and iii) species primarily breeding and foraging in nearby marine or coastal habitats (Moisan et al., 2023). The case of the red fox (*Vulpes vulpes*) was ambiguous. While the presence of breeding pairs has been confirmed in the study area (Lai et al., 2022), the extent of population establishment remains unclear and sightings are rare. Therefore, we decided to exclude this species.

Table 1: Species of the vertebrate community of Bylot Island and their corresponding migratory status (i.e., resident, partial migrant or migrant).

Functional group	Scientific name	English name	Migratory status
Ducks and loons	*Gavia pacifica*	Pacific loon	migrant
Ducks and loons	*Gavia stellata*	Red-throated loon	migrant
Ducks and loons	*Somateria spectabilis*	King eider	migrant
Ducks and loons	*Clangula hyemalis*	Long-tailed duck	migrant
Geese and swans	*Branta hutchinsii*	Cackling goose	migrant
Geese and swans	*Anser caerulescens*	Snow goose	migrant
Geese and swans	*Cygnus columbianus*	Tundra swan	migrant
Raptors	*Buteo lagopus*	Rough-legged hawk	migrant
Raptors	*Falco peregrinus*	Peregrine falcon	migrant
Raptors	*Bubo scandiacus*	Snowy owl	migrant
Ptarmigans	*Lagopus muta*	Rock ptarmigan	resident
Cranes	*Antigone canadensis*	Sandhill crane	migrant
Shorebirds	*Pluvialis dominica*	American golden-plover	migrant
Shorebirds	*Pluvialis squatarola*	Black-bellied plover	migrant
Shorebirds	*Charadrius hiaticula*	Common-ringed plover	migrant
Shorebirds	*Arenaria interpres*	Ruddy turnstone	migrant
Shorebirds	*Calidris canutus*	Red knot	migrant
Shorebirds	*Calidris melanotos*	Pectoral sandpiper	migrant
Shorebirds	*Calidris bairdii*	Baird's sandpiper	migrant
Shorebirds	*Calidris fuscicollis*	White-rumped sandpiper	migrant
Shorebirds	*Calidris subruficollis*	Buff-breasted sandpiper	migrant
Shorebirds	*Phalaropus fulicarius*	Red phalarope	migrant
Gulls	*Larus hyperboreus*	Glaucous gull	migrant
Jaegers	*Stercorarius longicaudus*	Long-tailed jaeger	migrant
Jaegers	*Stercorarius parasiticus*	Parasitic jaeger	migrant
Ravens	*Corvus corax*	Common raven	partial migrant
Passerines	*Eremophila alpestris*	Horned lark	migrant
Passerines	*Anthus rubescens*	American pipit	migrant
Passerines	*Calcarius lapponicus*	Lapland longspur	migrant
Passerines	*Plectrophenax nivalis*	Snow bunting	migrant
Lemmings	*Lemmus trimucronatus*	Nearctic brown lemming	resident
Lemmings	*Dicrostonyx groenlandicus*	Nearctic collared lemming	resident
Hares	*Lepus arcticus*	Arctic hare	resident
Ermines	*Mustela richardsonii*	American ermine	resident
Foxes	*Vulpes lagopus*	Arctic fox	partial migrant

b. Geography

The 389 km^2 study area is located on the southern plain of Bylot Island, Nunavut, Canada (72.889 N, -79.906 W; **Figure 4**). This area is characterized by a rolling plain, mostly from 0 to 300 m above sea level, located between mountains (up to 1900 m) to the north and east and the sea to the west and south.

c. Habitat

The study area comprises a combination of mesic tundra mainly on hills and gentle slopes (64 %), upland plateaus of sedimentary rock with drier/rockier habitat at higher elevation (20 %), low-lying wetlands interspersed with ponds (10 %) and larger bodies of water such as lakes and rivers (6 %; **Figure 3**). The vegetation of mesic habitat is characterized by prostrate shrubs (*Salix* spp., *Vaccinium uliginosum*, and *Cassiope tetragona*), forbs (*Luzula* spp., *Oxytropis maydelliana*, *Astragalus alpinus*, *Oxyria digina*, and *Polygonum viviparum*), grasses (*Arctagrostis latifolia* and *Poa arctica*) and mosses (e.g., *Aulacomnium* spp.; Gauthier et al. 2013, 2024b). Wetlands are found near water bodies (streams, ponds

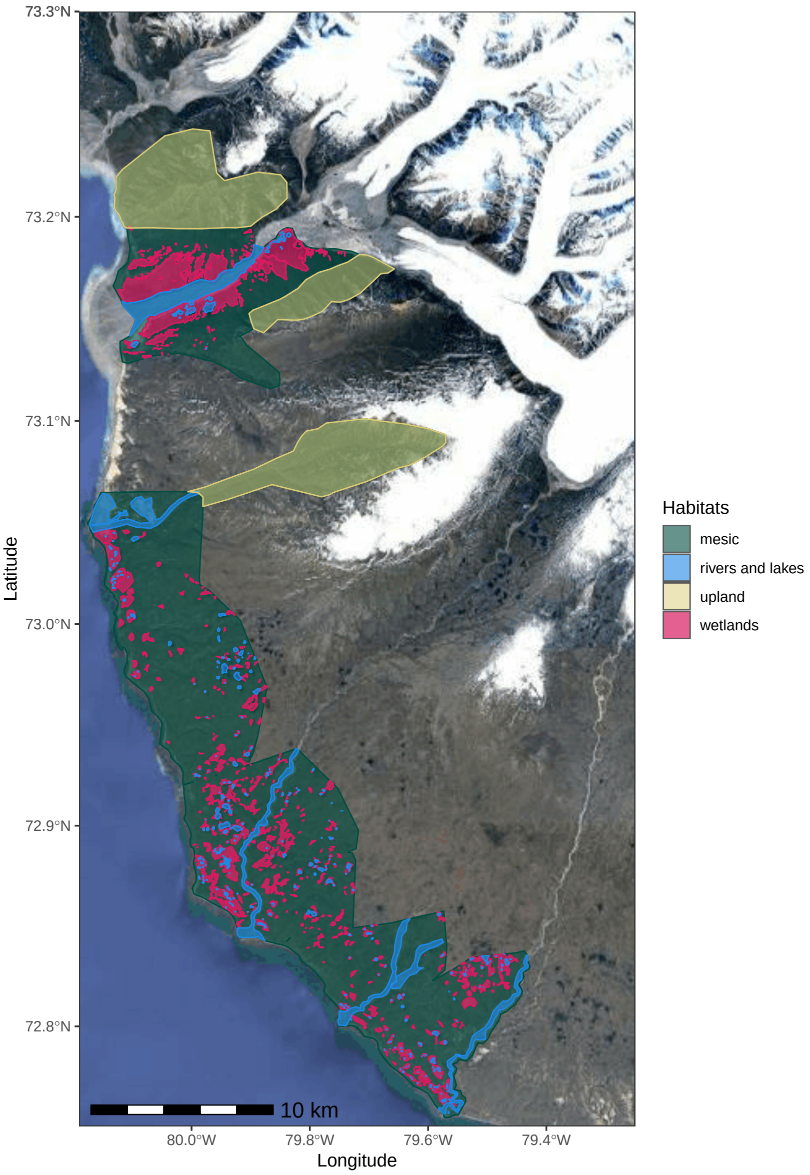

Figure 3: Map of the main habitat types within the study area, including mesic tundra, upland areas, wetlands, and aquatic environments (rivers and lakes).

and lakes) or in tundra polygons and present a vegetation typically associated with moss-covered fens with grasses and sedges (*Dupontia fisheri*, *Carex aquatilis*, and *Eriophorum scheuchzeri*; Gauthier et al. 2013, 2024b). See section *e. Hydrology* for details on wetlands and water bodies delineation. Upland habitat is drier and present a less abundant and rich vegetation with species such as *Dryas integrifolia* or *Saxifraga oppositifolia* (Gauthier et al., 2013, 2024b).

d. Geology

See Klassen (1993) for a detailed description of the geology of the study area.

e. Hydrology

Wetlands were delineated by photo-interpretation of high-resolution satellite images (30 cm; Louis-Pierre Ouellet, unpublished data). Lakes were delineated with aerial photos (see Corbeil-Robitaille et al. 2024), whereas rivers were delineated with google satellite images, resulting in a coarser delineation.

f. Site history

See Gauthier et al. (2024b,a) for a complete and detailed history of the site.

g. Climate

The mean annual air temperature since 1995 is -14.4°C, with mean seasonal temperature of 4.7°C in summer (June to August), -11°C in fall (September to November), -32.4°C in winter (December to February) and -19.4°C in spring (March to May; Centre of Northern Studies and Laval University 2019). The climate of the southern plain of Bylot Island is generally milder than that of the surrounding latitudes, as the plain present a southern exposure and the mountains to the north protect the plain from cold northerly winds (Gauthier et al., 2024b). In summer, the study area received on average 77.5 mm of precipitation. The study area typically remains free of snow from mid-June to late September with an average of 106 frost-free days annually (Gauthier et al., 2013). From September to December, snow depth typically increases continuously to reach about 15 cm (Centre of Northern Studies and Laval University, 2019). Snow depth is relatively constant from December to February and starts increasing again in March to peak in late May (∼27 cm) before decreasing until disappearance in mid-June (Centre of Northern Studies and Laval University, 2019). The summary statistics presented here on the climate of the South plain of Bylot Island are derived from automated weather stations located in the Qarlikturvik valley, which have been recording data on air temperature, thawing degree days, frost-free days, summer precipitation, snow thickness, and wind speed on a daily basis since 1992 (Centre for Northern Studies, 2024). Raw data from these weather stations are openly accessible at `https://nordicana.cen.ulaval.ca/fr/publication.php?doi=45039SL-EE76C1BDAADC4890`.

2. Experimental or sampling design

Research activities on Bylot Island take place from mid-May, well before the snowmelt, which occurs in early June, to August 20, in part due to logistical constraints in the latter case. The period therefore covers the arrival of migratory species and the entire breeding cycle for most species. The timing of surveys was adapted to the breeding cycle of individual species to maximize detection rates. However, the sampling period does not encompass the departure of some migratory species (e.g., loons, snowy owl and sandhill crane).

a. Permanent plots

The study area is divided into nine zones based on the sampling method and the level of field effort applied in each zone (**Figure 4**). Long-term monitoring of the community began in the 1990s in the Qarlikturvik valley (Gauthier et al., 2013, 2024b), which represents the zone of the study area with the highest annual sampling effort. Within the Qarlikturvik valley, the sampling is concentrated on the southern side of the glacial river (**Figure 5**), where the main research infrastructure is located. Another zone with extensive sampling efforts is Camp 2, located at the core of the snow goose colony, where the primary focus is to monitor snow goose nests. However, nests of many other avian species are also monitored within and around the snow goose colony in this zone. Camp 3, Pointe Dufour, Goose Point, and Malaview are zones where intensive sampling efforts are conducted annually, albeit for a relatively brief period (approximately one week) during the breeding season of most species (Gauthier et al., 2024b). The upland zones in the study area (defined as areas approximately 300 meters above sea level or more) are the Black Plateau, Southern Plateau, and Camp 3 Plateau. These zones are primarily visited to assess raptor nesting activity (Beardsell et al., 2016). The zone between the Qarlikturvik valley and Camp 3 received very little sampling effort and is therefore excluded from the study area.

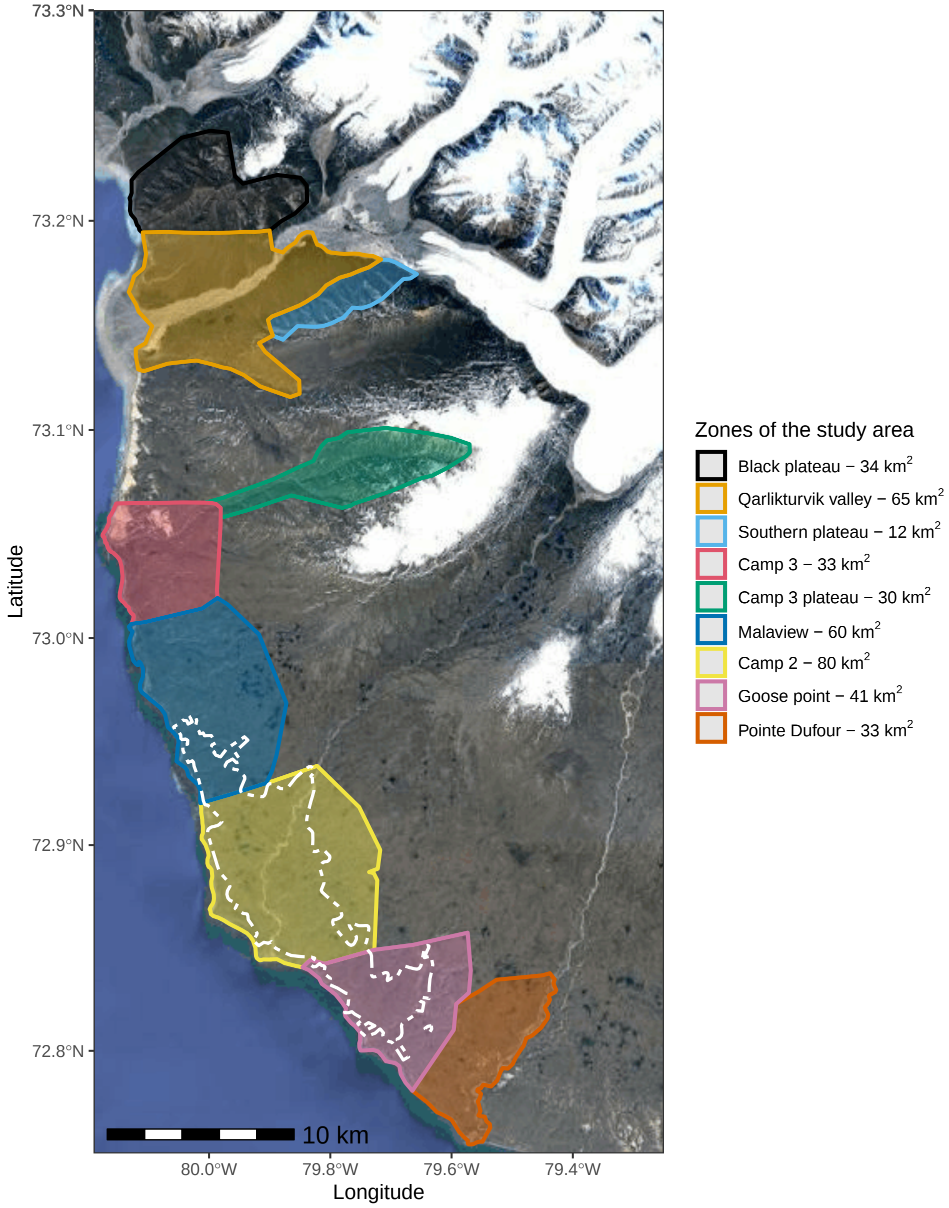

Figure 4: Map of the different zones (colored polygons) of the 389 km^2 study area located on the south plain of Bylot Island, Nunavut Canada. The perimeter of the snow goose colony is delineated by white dashes; we highlighted the perimeter in 2017 since it represents the average colony area (74 km^2).

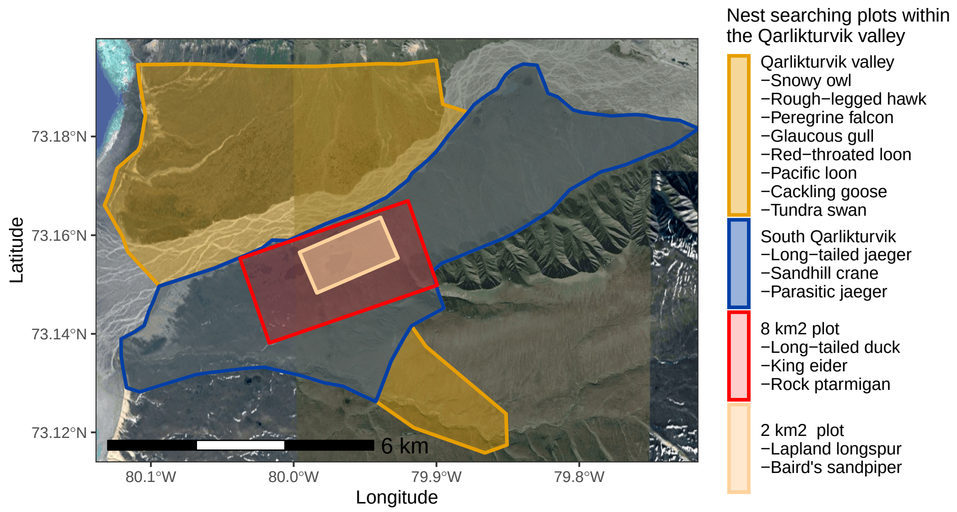

Figure 5: Intensive nests searching plots within the Qarlikturvik valley.

b. Avian nest monitoring

Avian nest monitoring was not conducted in 2020 and 2021 due to logistical constraints imposed by the COVID-19 pandemic. Systematic nest monitoring refers here to a systematic sampling approach aimed at documenting all nests within a specified area. Monitoring is considered opportunistic when there is a chance that some nests might not have been detected within a specific area. Nest densities derived from nest sampling could be underestimated due to early nest failure (i.e., failure that happened before our sampling period).

Pacific loon, red-throated loon, cackling goose, tundra swan and glaucous gull

Between 2004 and 2016, systematic searches for glaucous gull and cackling goose nests were conducted in the wetlands of the Qarlikturvik alley. Starting in 2017, nest searches in wetlands were carried out systematically across the entire study area. Since then, nest locations of other large wetland-nesting species—including tundra swans, red-throated loons, and Pacific loons—have also been recorded systematically, as these species nest in similar habitats (Duchesne et al., 2021; Gauthier et al., 2024b). Each year, all known or potential nesting sites were revisited. Observers detected nests by walking and scanning around ponds and lakeshores to identify any active nesting sites. These large species can be seen from a relatively long distance sitting on the nest or when flushing from the nest. Most of them (geese, swans and gulls) can also reveal their presence with alarm calls or nest de-

Table 2: Summary of vertebrate species monitoring in the Bylot Island study area. In this paper, we excluded certain years for specific species due to reduced sampling efforts. As a result, duration of times series presented here may differ slightly from those in Gauthier et al. (2024b). The annual density values are provided in the file BYLOT-species_density_monitoring.csv.

Species	Zone	Years	Number of years	Monitoring
Pacific loon	Whole study area	2017-2019, 2022	(4)	systematic
Red-throated loon	Whole study area	2017-2019, 2022	(4)	systematic
King eider	Qarlikturvik (8 km2 plot)	2005-2019, 2022	(16)	opportunistic
Long-tailed duck	Qarlikturvik (8 km2 plot)	2005-2019, 2022	(16)	opportunistic
Cackling goose	Qarlikturvik valley	2004-2019, 2022-2023	(18)	systematic
Cackling goose	Whole study area	2017-2019, 2022-2023	(5)	systematic
Snow goose	Camp 2	1999-2019, 2022-2023	(23)	systematic
Tundra swan	Whole study area	2017-2019, 2022	(4)	systematic
Rough-legged hawk	Qarlikt., Black & South plat.	2007-2019, 2022	(15)	systematic
Rough-legged hawk	Whole study area	2013-2019, 2022	(8)	systematic
Peregrine falcon	Qarlikt., Black & South plat.	2007-2019, 2022	(15)	systematic
Peregrine falcon	Whole study area	2013-2019, 2022	(8)	systematic
Snowy owl	Qarlikt., Black & South plat.	1996-2019, 2022-2023	(26)	systematic
Snowy owl	Whole study area	2012-2019, 2022-2023	(10)	systematic
Rock ptarmigan	Qarlikturvik (8 km2 plot)	2005-2019, 2022	(16)	opportunistic
Sandhill crane	South Qarlikturvik valley	2004-2019, 2022	(17)	opportunistic
Common-ringed plover	Whole study area	2015-2017	(3)	systematic
Baird's sandpiper	Qarlikturvik (2 km2 plot)	2005-2019, 2022-2023	(17)	systematic
Glaucous gull	Qarlikturvik valley	2004-2019, 2022	(17)	systematic
Glaucous gull	Whole study area	2017-2019, 2022	(4)	systematic
Long-tailed jaeger	South Qarlikturvik valley	2004-2019, 2022	(17)	systematic
Parasitic jaeger	South Qarlikturvik valley	2004-2019, 2022	(17)	systematic
Parasitic jaeger	Whole study area	2009-2019, 2022	(12)	opportunistic
Common raven	Whole study area	2013-2019, 2022	(8)	systematic
Lapland longspur	Qarlikturvik (2 km2 plot)	2005-2019, 2022-2023	(17)	systematic
Nearctic brown lemming	Qarlikturvik (trapping grids)	1995-2019, 2021-2022	(27)	systematic
Nearctic collared lemming	Qarlikturvik (trapping grids)	1995-2019, 2021-2022	(27)	systematic
American ermine	Whole study area	1993-2019	(27)	opportunistic
Arctic fox	Whole study area	2008-2016	(9)	systematic

fense displays. We are confident that nest detection probability was high for these species given the open landscape.

Snow goose

Snow geese nest in a large colony in the study area (**Figure 4**), but also in small aggregations distributed on the island, especially in years when snowy owls are nesting (Lepage et al., 1996; Reed et al., 2002). Since 1994, goose nests were systematically monitored on a 0.24 km^2 wetland at the center of the colony. Since 1999, nests were also systematically monitored on a variable number of plots, measuring 0.01 km^2 in wetland habitat and 0.04 km^2 in mesic habitat, randomly distributed throughout the goose colony (Gauthier and Cadieux, 2020a). The total area covered by the randomly distributed plots averaged 0.79 $\pm$0.37 km^2 per year. From 2010 onwards, except in 2020 and 2021, we opportunistically traced sections of the approximate boundary of the goose colony using a GPS receiver aboard a helicopter, taking advantage of regular flights across the study area whenever the flight path passed over the colony border (Duchesne et al., 2021).

Rough-legged hawk, peregrine falcon and common raven

Peregrine falcons, rough-legged hawks and common ravens nest on cliffs, near ravines, and on large rocky outcrops and tend to reuse the same nesting sites from one year to the next (Beardsell et al., 2016). Systematic monitoring of every known or potential nesting site has been carried out in the Qarlikturvik valley, Black plateau and Southern plateau since 2007 and throughout the study area since 2013 (Beardsell et al., 2016; Gauthier et al., 2020). Observers walked along ridges and scanned surrounding areas from vantage points to detect nesting birds. These large species can be seen from a relatively long distance sitting on the nest or when flushing from the nest. They can also reveal their presence with alarm calls or nest defense displays. We are confident that nest detection probability was high for these species. Each year the observers use slightly different paths to sample the areas, but locate the nests in the same positions, which supports a high probability of detection for these species. Most nesting sites were located in the upland zones of the study area, which include the Black Plateau, Southern Plateau and Camp 3 Plateau.

Snowy owl

Snowy owls predominantly nest in habitats similar to other raptors, favoring ridges in mountainous or hilly regions, although they can occasionally be found nesting on mounds in lowland areas (Seyer et al., 2020). Since 1996, searches for snowy owl nests have been conducted in the Black and Southern plateaus, as well as during searches for jaeger nests on the southern side of the glacial river in the Qarlikturvik Valley. Additionally, since 2012, nests have been recorded across the entire study area by scanning the landscape from hills and ridges during the nesting period (Duchesne et al., 2021). Given that snowy owls nest on elevated mounds, exhibit contrasting colors with the landscape, emit alarm calls, and display defensive behaviors, active nesting sites have a high probability of detection.

Long-tailed jaeger, parasitic jaeger and sandhill cranes

Since 2004, observers have walked parallel transects spaced 400 meters apart, covering the entire southern side of the glacial river in the Qarlikturvik Valley (33 km^2; **Figure 5**), during the nesting period. The aim of those transects was to record nests of long-tailed jaegers, parasitic jaegers, and sandhill cranes. Observers listened for alarm calls to detect territorial birds, and then located nests by observing the birds returning to their nests from elevated vantage points. We consider the sampling to be systematic for long-tailed and parasitic jaeger, since those species tend to leave their nest relatively far from the observer to perform mobbing behavior, and thus increasing their detection probability. We consider the sampling to be opportunistic for sandhill cranes, as individuals display defensive behaviors only at relatively close distances from their nests (see *Opportunistic nest monitoring* below).

Common-ringed plover

Between 2015 and 2019, observers conducted surveys of the primary nesting areas of the common-ringed plover. The survey involved walking in stony and sandy shores and gravel bars with scarce vegetation along rivers. Nests were found by detecting individuals exhibiting reproductive behaviors, such as incubation, alarm calls, or distraction displays. The sampling effort was particularly intensive between 2015 and 2017. Small areas along the coast or on the banks of smaller rivers that could potentially serve as nesting sites may have been overlooked.

Lapland longspur and Baird's sandpiper

Since 2005, nests of passerines and sandpipers have been extensively monitored across an 8 km^2 (4x2 km) area in the Qarlikturvik valley. We considered the sampling to be most systematic within a core 2 km^2 (2x1 km) plot in this area (**Figure 5**). We excluded relatively large water bodies (0.26 km^2) to calculate nest density in the plot due to the presence of a large lake, which leaves an area of 1.74 km^2 available for nesting. An observer conducted systematic searches of this plot during the entire breeding season to locate and monitor as many passerine and shorebird nests as possible. Assuming the observer can detect all nests within a 5 or 10 meter radius, analysis of daily GPS tracks shows that the observer covered a minimum area of 0.72 $\pm$0.12 (5 m) or 1.09 $\pm$0.17 km^2 (10 m) of the core area annually (n= 3 years). Additionally, several other observers conducting related field work in the same zone reported all passerine and shorebird nests found opportunistically.

Opportunistic nest monitoring

Since 2005, we also noted the nest location of any other bird species encountered opportunistically during travel or while carrying out the protocols for the previously described species. The sampling was particularly intensive in the defined 8 km^2 area in the Qarlikturvik valley. The accuracy of nest monitoring in this plot thus depends on the species detection probability. We are confident to obtain a realistic order of magnitude for the number of nests present for relatively large bodied species in this area (i.e., sandhill crane, rock ptarmigan, long-tailed duck and king eider). Additionally, starting in 2009, a significant effort has been made each year, though not systematically, to visit known nesting territories of parasitic jaegers throughout the study area.

c. Observation of individuals

Vertebrate count transects

From 2010 to 2023, observers walked 500-meter linear transects where all vertebrate individuals observed within 150 meters on either side were counted (146 to 320 transects per year). Transects were distributed across all lowland zones of the study area, typically in mesic habitat, and were carried out during the nesting period (between June 21 and July 14; Lamarre et al. (2017); Duchesne et al. (2021); **Figure 6**). As a calibration exercise, all observers were trained to estimate a distance of 150 m using a rangefinder prior to data collection. Furthermore, specifically for American golden-plovers, we measured the distance of each observed individual to the transect path. Observations of species considered as occasional visitors (i.e., species lacking confirmed breeding occurrences on the study site, or species observed solely within a single year, or species primarily breeding and foraging in nearby marine or coastal habitats) were removed.

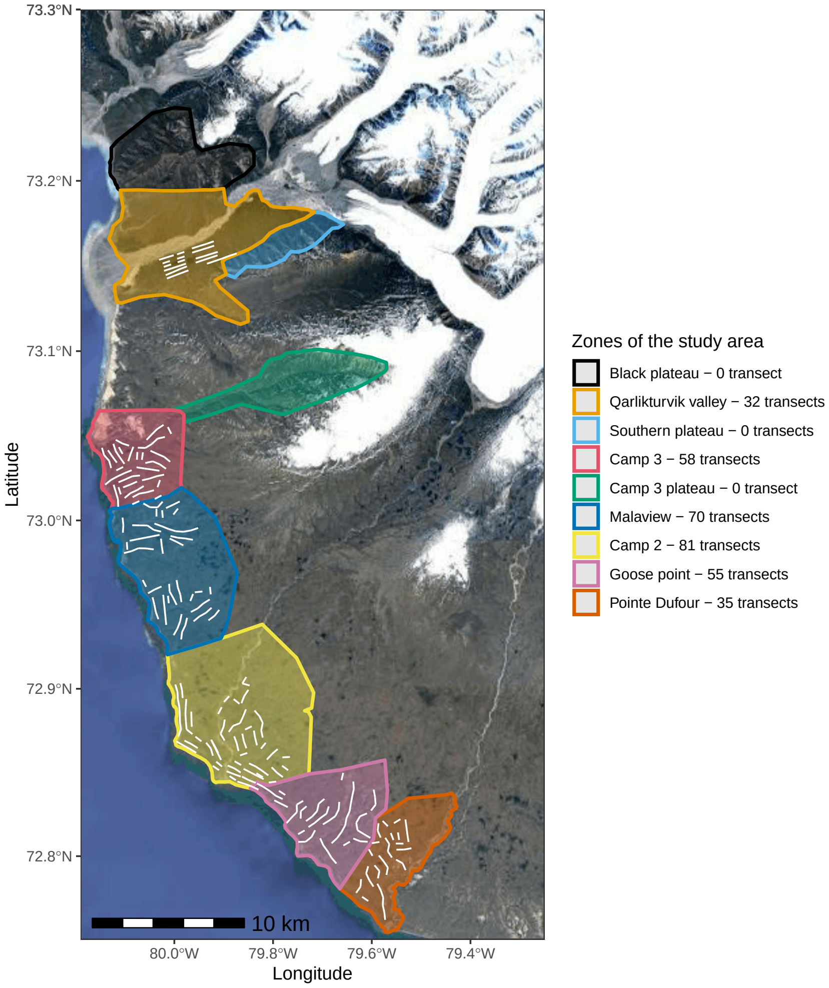

Figure 6: Spatial distribution of vertebrate count transects (white lines) on the south plain of Bylot Island (146 to 320 transects per year).

Snow goose point count

At the start, middle, and end of each vertebrate count transect, a point count with a radius of 125 meters was conducted to determine the number of snow goose breeding pairs. On average, 613 $\pm$142 point counts were sampled each year, covering an area of 30 $\pm$7 km^2. Observers were trained with a rangefinder to estimate a distance of 125 m prior to data collection.

Incidental observations

Since 2007, observers have recorded all vertebrate species observed opportunistically during field work and tallied the total number of individuals at the end of each day (Gauthier and Cadieux, 2020b; Gauthier et al., 2024b). Observations are made from mid-May to late August, but the effort is highest during late June and early July. The number of hours spent in the field served as a proxy for the sampling effort. Observations of species considered as occasional visitors (i.e., species lacking confirmed breeding occurrences on the study site, or species observed solely within a single year, or species primarily breeding and foraging in nearby marine or coastal habitats) were removed (see Gauthier et al. 2024b for a comprehensive list of all species observed in the study area over the years). The original data, as well as any future updates, can be accessed at: `https://nordicana.cen.ulaval.ca/fr/publication.php?doi=45645CE-A24D883A6676492E`. We used the number of individuals observed per hour spent in the field calculated by Gauthier et al. (2024b) as an index of relative abundance for each species. Moreover, we separated observations made in lowland from those in upland zones to have a relative abundance of each species in each of these two broad categories (**Table 3**). Given that incidental observations lacked georeferencing, we opted to extract upland observations by focusing on observations made during visits to rough-legged hawk nests, which are mostly located in upland areas.

Table 3: Index of relative abundance (i.e., number of individuals observed per hour) derived from incidental daily observations for selected vertebrate species in lowland (i.e., Qarlikturvik valley, Camp 3, Malaview, Camp 2, Goose point and Pointe Dufour) and upland (i.e., Black plateau, Southern plateau and Camp 3 plateau) zones of the Bylot Island study area. The ratio compares relative abundance indexes between these two types of zones, calculated by dividing the upland by the lowland index of relative abundance.

	Individuals/hour		
Species	Upland	Lowland	Ratio
Rock ptarmigan	0.03	0.03	1
Sandhill crane	0.42	0.287	1.5
American golden-plover	0.26	0.394	0.7
Black-bellied plover	0.02	0.032	0.6
Ruddy turnstone	0.01	0.007	1.3
Red knot	0.00	0.033	0
Pectoral sandpiper	0.02	0.034	0.6
Baird's sandpiper	0.31	0.32	1
White-rumped sandpiper	0.04	0.137	0.3
Buff-breasted sandpiper	0.00	0.001	0
Red phalarope	0.01	0.038	0.2
Horned lark	0.24	0.154	1.6
American pipit	0.34	0.024	14.2
Lapland longspur	1.93	2.641	0.7
Snow bunting	0.59	0.092	6.4
Arctic hare	0.02	0.009	2

Testimonials of ermine sightings

There was no direct estimation of ermine abundance on Bylot Island as they are quite difficult to obtain. The density estimates for ermine were derived from an annual abundance index established by Bolduc et al. (2023), which relied on testimonials provided by observers across the whole study area from 1993 to 2019. The testimonials provided by observers were used to create an abundance index ranging from 0 to 3 (Bolduc et al., 2023). In this index, a score of 0 corresponds to the absence of ermine sightings, 1 indicates a single sighting of a lone individual, 2 represents multiple sightings of lone individuals, and 3 signifies at least one sighting of a family group. Scores of individual participants were averaged annually as detailed in Bolduc et al. (2023).

d. Capture of individuals

Lemming trapping

Since 2004, Nearctic brown and collared lemmings were live-trapped 3 times during the summer (mid-June, mid-July, and mid-August) in two 11 ha grids. Each grid is made of 144 traps separated by 30 m according to a cartesian plane, one in mesic habitat and the other in wet habitat, located in the Qarlikturvik valley (Fauteux et al., 2015; Gauthier, 2020). Density of each species was estimated at each occasion using spatially explicit capture-recapture methods (Fauteux et al., 2015). From 1995 to 2016 snap-trapping was performed once a year (mid-July) along 2 groups of transects located in the same habitats than the trapping grids (Gruyer et al., 2008).

Abundance indices derived from snap-trapping (i.e., number of animals caught per 100 trap-nights) were converted to density estimates using a linear model based on data from 2004 to 2016, when both snap-trapping and live-trapping were conducted concurrently (Fauteux et al., 2018). Separate regressions were performed for mesic and wetland habitats. Both live-trapping densities and snap-trapping indices were log-transformed to reduce large variations and improve model fit.

In some years, no lemming was captured on our trapping grids. However, this does not mean that lemmings were totally absent from the study area when extrapolating to the landscape level. In those years, we thus replaced zeros by half of the lowest lemming density that our sampling scheme could have detected, which corresponded to a situation where only a single lemming would have been captured. Prior to 2004, half of the minimum detectable density was 0.07 lemming/ha and after that, 0.025 lemming/ha (the difference was due to the change in trapping method in 2004). Therefore, zeros were replaced by these values in the dataset.

Arctic fox movement tracking

In order to assess fox abundance based on the size of their home range, 109 Arctic foxes were fitted with Argos Platform Transmitter Terminals mounted on collars between 2008 and 2016 (Lai et al., 2015; Christin et al., 2015; Dulude-de Broin et al., 2023). Foxes were captured between May and August across the study area, within and outside the goose colony (Dulude-de Broin et al., 2023). Sampling of animal locations was set for an interval of 1 or 2 days and only locations between May 1 and October 30 were retained (Dulude-de Broin et al., 2023).

Parasitic jaeger banding

In 2009, a significant effort was made to band as many parasitic jaegers as possible within the study area. This effort resulted in the banding of 17 adult individuals (Therrien and Gauthier, unpublished data).

e. Species body mass

All vertebrate individuals captured for marking purposes were systematically weighed: snow goose (G. Gauthier, M.-C. Cadieux and J. Lefebvre, unpublished data), snowy owl (Therrien et al., 2012; Robillard et al., 2018), American-golden plovers (Lamarre et al., 2021), common-ringed plovers (Léandri-Breton et al., 2019), other shorebirds (J. Bêty, unpublished data), glaucous gulls (Gauthier et al., 2015), long-tailed jaeger (Seyer et al., 2019), parasitic jaegers (J.-F. Therrien and G. Gauthier, unpublished data), Lap-

land longspurs (J. Bêty and G. Gauthier, unpublished data), lemmings (Gauthier, 2020), American ermine (Bilodeau and Bolduc, unpublished data) and Arctic foxes (Lai et al., 2015). We retrieved the mean body mass of cackling geese captured and banded on Baffin Island presented in Neufeld (2021). When not available, we extracted mean body mass from the literature (Wilman et al., 2014).

3. Research methods

a. Field/laboratory

The methods used to estimate annual or mean species abundance at the landscape scale are summarized in **Table 4**.

Pacific loon, red-throated loon, cackling goose, tundra swan and glaucous gull

Based on the systematic and intensive search for the glaucous gull, cackling goose, tundra swan, red- throated loon and Pacific loon nests in wetlands, we are confident that we have found nearly all nests across the study area from 2017 to 2019 and in 2022. We observed a relatively strong correlation between the nest density of glaucous gulls in the Qarlikturvik valley and the nest density across the entire study area (R^2= 0.7, p = 0.16, n= 4). Consequently, we estimated the density of glaucous gulls at the scale of the study area between 2004 and 2016 based on the nest density in the Qarlikturvik valley ($y = 0.12409x + 0.13774$). We used a similar approach for cackling geese with the following equation $y = 0.60236x + 0.02019$ considering the strong correlation observed between the nest density in the Qarlikturvik valley and the nest density across the entire study area (R^2= 0.75, p = 0.06, n= 5). The first cackling goose nest was found in the study area in 1996. Since then, the total number of nests found annually appears to have increased exponentially (**Figure 7**). It is important to note that the substantial increase in monitoring effort from 2017 onward may have influenced the observed trend in nest numbers. Nest monitoring of loons and swans was not considered systematic in the Qarlikturvik valley before 2017, therefore we did not extend the time series for those species. Since annual abundance estimates for these species are derived from nest monitoring, they reflect only breeding individuals.

Snow goose

Between 1999 and 2023, we assessed the abundance of snow geese in the study area through a multi-step process. We calculated the mean annual density of snow goose nests separately in the mesic and wetland habitats of the area occupied by the goose colony annually. We made slight adjustments to the goose colony perimeter defined from helicopter flights to include all snow goose point counts where at least one breeding pair had been observed (**Figure 8**). To determine the mean density of nesting geese in wetlands, we divided two times (assuming two individuals per nest) the total number of nests found during systematic nest searches by the total area of wetlands sampled. Systematic nest search plots cover in average 4% of the wetland habitat within the goose colony. The density of geese nesting in mesic

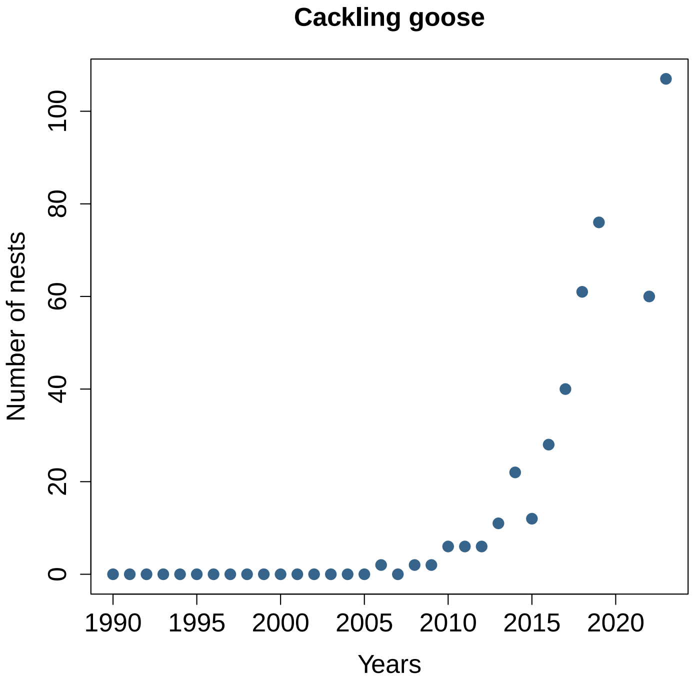

Figure 7: Number of cackling goose nests found across the study area over time. The first nest was found and 1996 and nest monitoring effort increase substantially across the study area since 2017.

habitat, a less preferred nesting habitat (Lecomte et al., 2008), was averaged from three independent methods: systematic nest searches, vertebrate count transects, and snow goose point counts; **Figure 9**). Systematic nest searches were highly precise, but covered a relatively small area (on average, 1% of the mesic habitat within the goose colony), whereas transects and point counts were less precise but covered larger areas, averaging 20% and 12% of the mesic habitat, respectively. For each method, we calculated the mean density of breeding individuals in mesic habitat by dividing the number of birds (or nests) recorded by the area sampled. We found a strong and statistically significant correlation between density estimates derived from vertebrate count transects and point counts (Sperman's rank correlation r_s= 0.8; p=0.003). However, there was no significant correlation between density estimates from nest sampling plots and either the transect data (r_s=0.37; p=0.24) or the point counts (r_s=0.22; p= 0.5). Despite the lack of correlation between observations (transects and point counts) and nest sampling, we chose to average the estimates from all three methods since observations and nest monitoring present complementary strengths and limitations. Transects and point counts offer broader spatial coverage but lower precision, while nest sampling provides higher precision but with a more restrained spatial coverage. Averaging the three methods allows us to balance these trade-offs and produce estimates that are less precise individually, but likely more accurate overall. Lastly, to transform the densities in total abun-

dance, we determined the annual proportion of wetland and mesic habitats within the goose colony and multiplied the area of each habitat by the density of breeding individuals. For the period 1999 to 2009, we used the average limits of the colony over the period 2010 to 2023 because we did not conduct aerial survey of the colony. Moreover, during the same period, nest density in the mesic habitat was derived only from systematic nest search (**Figure 9**). All annual estimates of snow goose abundance represent breeding individuals.

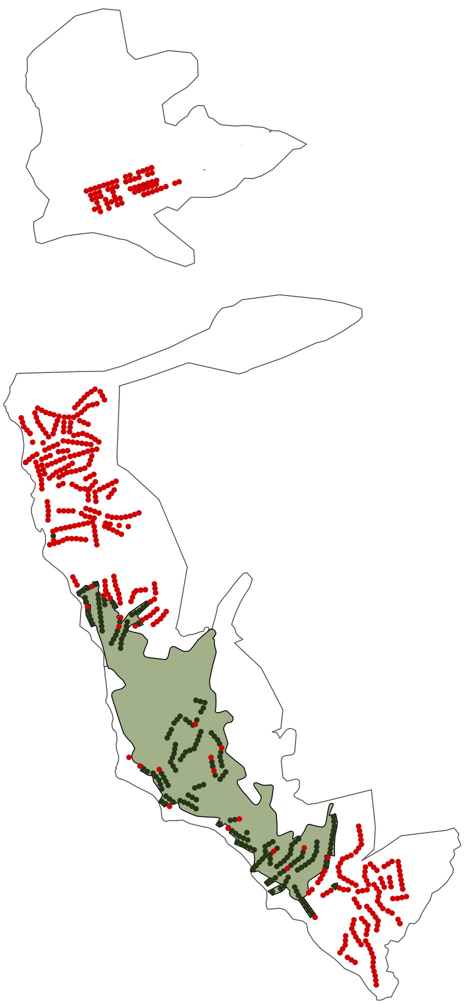

Figure 8: Map showing the region occupied by the snow goose colony in 2017 (green polygon) as an example. The perimeter was first defined opportunistically using a GPS receiver aboard a helicopter, taking advantage of regular flights across the study area whenever the flight path passed over the colony border. The perimeter was then slightly adjusted to include all snow goose point counts where at least one breeding pair had been observed in that year (green dots). Snow goose point counts where no breeding geese were observed in that year are presented as red dots.

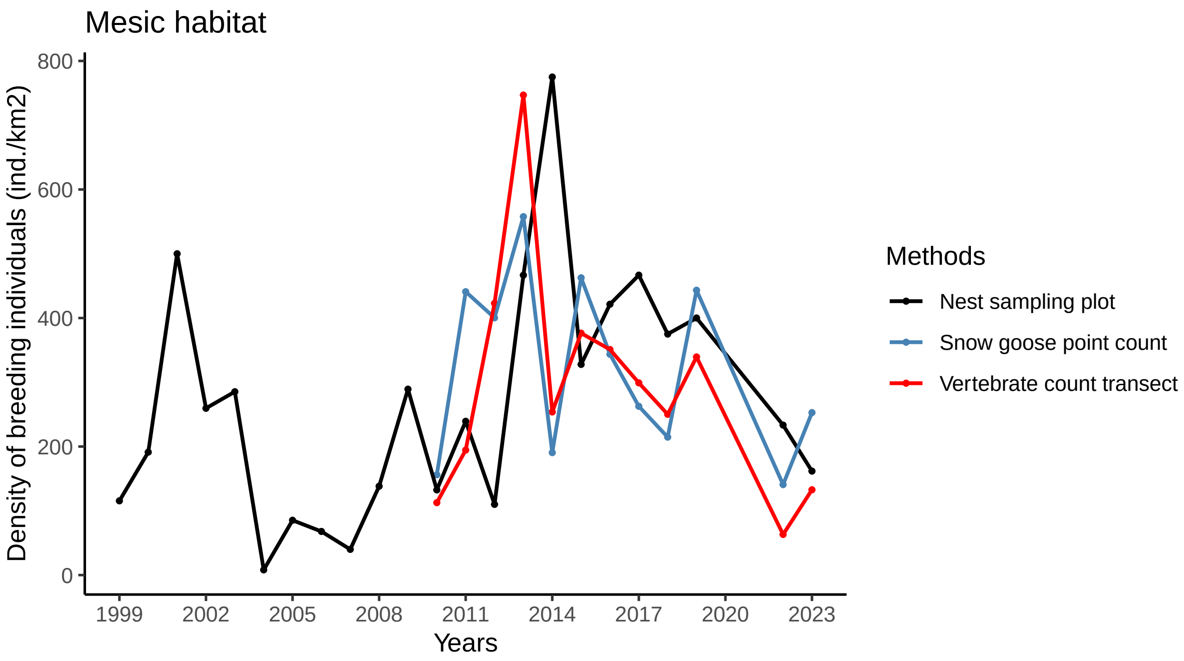

Figure 9: Estimates of breeding goose density in mesic habitat within the Bylot Island snow goose colony using three independent methods: Nest sampling plot, Snow goose point count, Vertebrate count transect.

King eider and long-tailed duck

We first estimated the abundance of both king eiders and long-tailed ducks at the scale of the study area based on the annual nest density of each species found in the 8 km^2 extensive nest search area located in the Qarlikturvik valley. We extrapolated the mean nest density in the wetlands of the Qarlikturvik valley to the wetlands of the study area (38.35 km^2). We transformed nest density to abundance of breeding individuals by multiplying it by a factor of two (assuming two individuals per nest). We acknowledge that the opportunistic monitoring of these species likely underestimated their true nest density. However, considering the extensive sampling effort deployed annually within this area, we are confident to obtain a realistic order of magnitude for the number of nests present. Because duck sightings are frequent throughout the breeding period, yet only a few nests are found, we believe there may be a significant portion of non-breeding individuals. Therefore, we employed an additional method to estimate the overall duck populations without differentiating between breeding and non-breeding individuals. As an alternative approach, we estimated the abundance of ducks based on the indices of relative abundance (i.e., the number of individuals observed per 100 hours) presented by Gauthier et al. (2024b). We assumed that the ratios between relative and actual abundance are the same (i.e., similar detection probability) in duck and loon species. We therefore derived the absolute abundance of long-tailed ducks and king eiders from their relative abundances using the ratio between relative and absolute abundances of

red-throated loons as a reference. In summary, duck abundance estimates based on nest monitoring reflect only breeding individuals, while those based on observations may include non-breeding individuals.

Rough-legged hawk, peregrine falcon and snowy owl

We estimated the abundance of breeding rough-legged hawks, peregrine falcons and snowy owls based on systematic nest monitoring conducted throughout the study area for these species. To convert the number of nests into breeding abundance, we multiplied it by two (assuming two individuals per nest). For snowy owls, we extended the time series from 1996 to 2011 based on a linear regression between nest density in the Qarlikturvik valley and nearby plateaus (Black and Southern plateaus) and nest density across the entire study area ($y = 0.68867x - 0.00173$; $R^2 = 0.98$; $p < 0.0001$, n= 10). The linear regression included only three years in which snowy owls were present. To avoid overestimating the strength of the correlation, we restricted the analysis to those years and still observed a strong relationship despite the very small sample size ($R^2 = 0.98$, p= 0.096, n= 3). We used the same approach for rough-legged hawks ($y = 0.49851x$, $R^2 = 0.99$, $p < 0.0001$, n= 8) to extend the time series from 2007 to 2012. As with snowy owls, the correlation remained strong when considering only the years in which hawks were present ($R^2 = 0.98$, $p < 0.0001$ n= 7). We did not extend the time series for peregrine falcons because the correlation was not as strong ($R^2 = 0.20$, p= 0.27, n=8). Since annual abundance estimates for these species are derived from nest monitoring, they reflect only breeding individuals.

Rock ptarmigan

We estimated the abundance of rock ptarmigans based on the mean annual nest density measured in the 8 km^2 extensive nest search area of the Qarlikturvik valley. While we acknowledge that the opportunistic monitoring of this species likely underestimates nest density, the extensive sampling effort deployed annually within this area gives us confidence in obtaining a realistic number of nests. We then extrapolate the density to the whole study area, without distinction between mesic, wetland and upland habitats (**Table 3**). Among the 6 nests found in the study area, 4 were located in mesic habitat, while one nest was found in a wetland and another in an upland habitat. Nest site selection by rock ptarmigan has not been well documented in the Canadian Arctic; however, a study conducted at Sarcpa Lake (n= 7 nests) also highlight that rock ptarmigans can nest in a variety of habitats (Montgomerie et al., 1983). To convert the number of nests into abundance, we multiplied it by two (assuming two individuals per nest). Since the estimate of rock ptarmigan abundance is based on nest monitoring, it reflects only breeding individuals.

Sandhill crane

We determined the mean nest density of sandhill cranes from the nest sampling between 2004 and 2023 on the southern side of the glacial river in the Qarlikturvik valley. We determined mean nest density by dividing the mean annual number of nests recorded by the area of the surveyed zone (33 km^2). We estimated total abundance by multiplying mean nest density by the total area of the study area. We assumed a uniform density across the study area, as sandhill crane nests have been observed in wetland, mesic, and upland habitats. Since the estimate of sandhill crane abundance is based on nest monitoring, it reflects only breeding individuals.

American golden-plover and black-bellied plover

We used a distance sampling approach to estimate the abundance of American golden-plovers in the lowland zones of the study area between 2014 and 2023. Observations of plovers were made along vertebrate count transects mainly in mesic habitat. Perpendicular distance between detected individuals and the transect path were used (n= 1015) to estimate a detection function with the *ds* function from the *Distance* package (Miller et al., 2019). To determine the detection function, we applied a truncation distance of 150 m (i.e., maximum distance on either side of the observer where observations have been considered) and selected the model with the lowest AIC, which included a "hn" key and a single "cos" adjustment term. We excluded observations of groups with more than four individuals, as these likely indicated groups of non-breeders. We did not estimate abundance in wetland habitat because American golden-plovers nest almost exclusively in mesic habitat (Parmelee et al., 1967). We estimated the abundance in the upland zones (i.e., plateaus) by applying a correction factor to the abundance in lowland zones. This correction factor was based on the relative abundance ratio between the upland and lowland zones (**Table 3**). Since groups of non-breeding individuals (>4 individuals) were excluded from the analysis, the estimates represent the abundance of breeding individuals.

To determine the abundance of black-bellied plovers, we used the mean number of black-bellied plovers and American golden-plovers observed per transect as an index of relative abundance. For both species, observations of groups with more than four individuals were removed. We assumed that the ratios between relative and actual abundance are the same (i.e., similar detection probability) among those species. This assumption is realistic as those species present similarities in size, color, and reproductive behavior. We therefore derived the absolute abundance of black-bellied plovers from their relative abundance using the ratio between relative and absolute abundances of American golden-plover as a reference. As an alternative approach to determine black-bellied plover abundance, we used the same approach as previously described, but with the indices of relative abundance presented by Gauthier

et al. (2024b), which was derived from incidental daily observations. The estimate of abundance derived from transect observations reflects breeding individuals, as groups of non-breeding individuals (>4 individuals) were excluded. However, the estimate based on incidental observations may include non-breeding individuals.

Common-ringed plover

To estimate the abundance of common-ringed plovers in the study area, we relied on the total number of nests recorded annually from 2015 to 2017, during which the primary nesting sites underwent intensive sampling. We multiplied the total nest count by two (assuming two individuals per nest). Since abundance estimates for the common-ringed plover are based on nest monitoring, they reflect only breeding individuals.

Lapland longspur and Baird's sandpiper

We estimated the abundance of Lapland longspurs in the different lowland zones of the study area based on a relation between nest density and the number of individuals observed per transect (**Figure 10**). For Baird's sandpiper, we employed a similar approach, but instead of using the mean number of individuals observed per transect, we used the mean proportion of transects where at least one individual was detected. We made this adjustment because this species was less frequently observed. Observations of Baird's sandpiper groups containing more than four individuals were removed to exclude potential non-breeding individuals. In this relationship, nest density for these two species came from the intensive nest sampling conducted within the core 2 km^2 area of the Qarlikturvik valley and observations of individuals from transects carried out in the larger 8 km^2 area in which the core area was located (**Figure 5**). This approach allowed us to incorporate a larger sample size from the transects while focusing on a measure of nest density determined systematically. Transects observations in lowland were then converted into nest density using the regressions equation, and then in total number of individuals by multiplying by the area and a factor 2. We estimated the density of both species in the upland zones by applying a correction factor to the annual mean density in lowland zones. This correction factor was based on the relative abundance ratio between the upland and lowland zones (**Table 3**). We acknowledge that the relation for Baird's sandpiper is weaker. However, incorporating spatial information derived from transect observations should offer some refinement and result in more accurate estimates compared to assuming uniform density across zones (which is a common assumption in food web modeling studies). Since abundance estimates for these species are based on a correlation between observations and nest density, they reflect the abundance of breeding individuals.

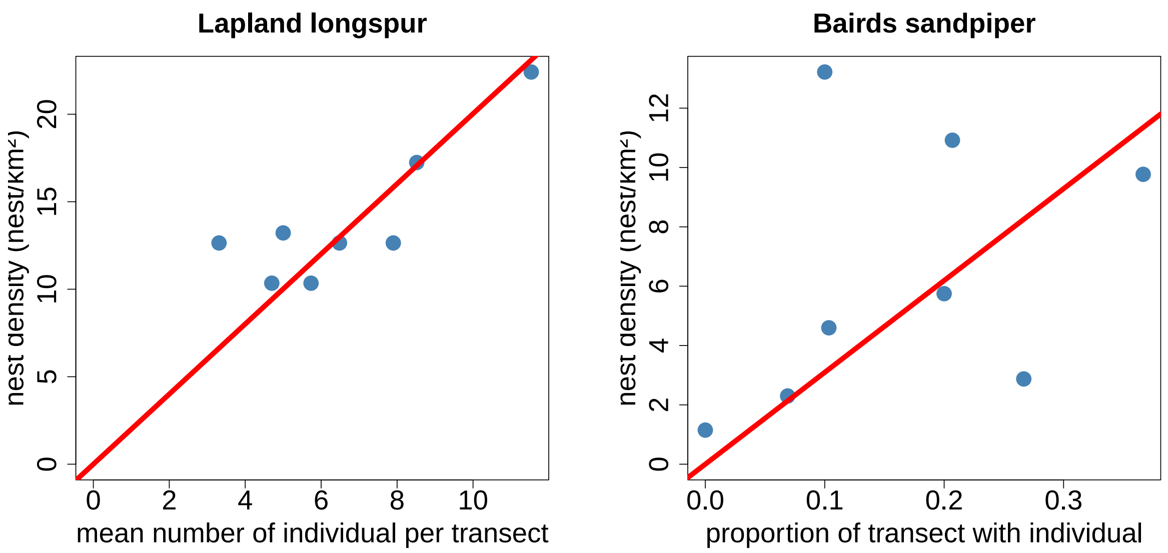

(a) (b)

Figure 10: a) The red line shows the linear relation between nest density and the number of individuals observed per transect, described by the equation nest density $= 2.0031 \times$ number of individuals per transect. The relation was forced through the origin. Blue dots represent annual values (2014–2019 and 2022–2023) of nest density and mean number of individuals per transect from the core sampled area of the Qarlikturvik Valley. We assumed that the relation between nest density and observations on transects is linear. b) The red line shows the linear relation between nest density and the proportion of transects with at least an individual, described by the equation nest density= 30.9519 x proportion of transects with at least one individual. The relation was forced through the origin. Blue dots represent annual values (2014–2019 and 2022–2023) of nest density and mean proportion of transects with at least an individual from the core sampled area of the Qarlikturvik Valley. We assumed that the relation between nest density and observations on transects is linear.

Other passerines and sandpipers

We estimated the abundance of other passerines (horned lark, American pipit, and snow bunting) in the lowland zones of the study area with the regression equation between number of individuals per transect and nest density of the Lapland longspur (see section *Lapland longspur and Baird's sandpiper*). We assumed here a similar detection probability for all species. We used the same approach for other sandpiper species (white-rumped sandpiper, pectoral sandpiper, buff-breasted sandpiper, red knot, ruddy turnstone and red phalarope) based on the regression equation for the Baird's sandpiper (see section *Lapland longspur and Baird's sandpiper*). For all these species, we estimated the density in the upland zones by applying a correction factor to the mean density in lowland zones. This correction factor was based on the relative abundance ratio between the upland and lowland zones (**Table 3**). Nest density was then converted in number of individuals by multiplying by the

area and a factor 2. As an alternative approach, we estimated the abundance of other passerines and sandpipers based on the indices of relative abundance (i.e., the number of individuals observed per 100 hours) presented by Gauthier et al. (2024b). We assumed that the ratios between relative and actual abundance are the same (i.e., similar detection probability) among both passerine and sandpiper species. We therefore derived the absolute abundance of other passerine and sandpiper species from their relative abundances using respectively, the ratios between relative and absolute abundances of Lapland longspur (passerines) and Baird's sandpiper (sandpipers) as references. For passerines, we assumed that all individuals observed were breeding, so abundance estimates represent breeding individuals. For shorebirds, observations of groups larger than four individuals were excluded from transects, meaning transect-based estimates represent breeding individuals. However, estimates derived from daily incidental observations may include non-breeding individuals.

Long-tailed jaeger

We determined the annual nest density of long-tailed jaegers from the systematic nest sampling between 2004 and 2023 on the southern side of the glacial river in the Qarlikturvik valley. We determined nest density by dividing the annual number of nests recorded by the area of the surveyed zone (33 km^2). As long-tailed jaegers typically nest in mesic habitat (Andersson, 1971), we multiplied the area occupied by mesic habitat across the study area by the nest density obtained in the surveyed zone and by two to obtain the total abundance of individuals (assuming two individuals per nest). Since abundance estimates for the long-tailed-jaeger are based on nest monitoring, they reflect only breeding individuals.

Parasitic jaeger

Based on the opportunistic nest monitoring of parasitic jaegers across the study area, an average of 4 nests is found annually, a small number considering that parasitic jaegers were frequently observed at the study site (Gauthier et al., 2024b). This suggests that there may be non-breeding individuals present at the study site, or alternatively, individuals may regularly travel long distances, potentially from outside the study area, to forage during the breeding season. Due to limited data availability for estimating the abundance of non-breeding parasitic jaegers, we relied on the maximum number of adults banded (breeding and non-breeding) during a single year (17 individuals in 2009; Therrien, unpublished data) as the minimum abundance on the study area. This corresponds to a density of 0.04 individuals/km^2. For comparison, Taylor (1974) measured a density of 0.06 individual/km^2 on Bathurst Island.

Common raven

Despite the intensive nest searches for raptors in upland zones, we never found more than one common raven nest each year, a small number considering the frequent raven observations at the study site (Gauthier et al., 2024b). This indicates the potential presence of non-breeding individuals or individuals that breed outside the study area but use it for foraging throughout the breeding period. Therefore, we opted for alternative approaches based on individual counts to estimate the abundance of both breeding and non-breeding ravens. As a first approach, we based our estimate of ravens on the number of glaucous gulls observed per transect. We assumed that the ratios between relative and actual abundance are the same (i.e., similar detection probability) among those species. This assumption is reasonable as those species present similarities in size and foraging strategy. We therefore derived the absolute abundance of common ravens from their relative abundance using the ratio between relative and absolute abundances of glaucous gulls as a reference. Independently, we estimated the abundance of common ravens with the same approach but using the indices of relative abundance presented by Gauthier et al. (2024b), which was derived from incidental daily observations, rather than observations from the transects. Potential non-breeding individuals are included in all estimates of common raven abundance.

Nearctic brown and collared lemming

Between 1995 and 2003, we used the density estimates derived from the snap-trapping indices obtained in late July in each habitat. Between 2004 and 2007, annual abundance of each lemming species was based on the late-July density estimates on trapping grid in wet and mesic habitats. However, starting from 2008, estimates were derived from the mean density recorded in mid-July and mid-August, except for two instances: 2019 and 2021. In 2019, due to an exceptionally early snowmelt and thus an early decline in lemmings during the summer, we only retained value from mid-July. In 2021, we relied solely on data gathered in August because it was the only trapping period carried out that year. To scale the estimated densities from the wet and mesic grids to the entire study area, we used the proportions of mesic habitats (64%) and wet habitats (10%) measured within the study area. Lemming abundance estimates include juveniles and therefore reflect both breeding and non-breeding individuals.

Arctic hare

Arctic hares are primarily observed in the upland zones of the study area, where sampling effort is limited. We thus derived abundance of hares from the estimated abundance of Arctic foxes based on indices of relative abundance presented in (Gauthier et al., 2024b), which were derived from incidental daily observations. We dou-

bled the density of Arctic hares in the upland zones (i.e., plateaus), as twice as many individuals were observed per hour of fieldwork there compared to lowland zones (**Table 3**). However, it is worth noting that assuming a similar detection probability between foxes and hares might lead to an overestimation of hare detection probability due to behavioral differences between the species. Therefore, we most likely underestimate the actual abundance of Arctic hares in the study area. Breeding and non-breeding hares are not distinguished in incidental observations; therefore, the abundance estimate may include non-breeding individuals.

American ermine

We estimated the annual abundance of ermines by transforming the annual index of relative abundance provided in Bolduc et al. (2023) into individual density. Annual values ranged from 0, indicating no ermine sighting, to 2.88, which signifies that nearly all observers observed at least one family group during their field season. We independtly obtained measures of minimum (0.02 ind./km^2) and maximum (0.4 ind./km^2) ermine density, which were determined from estimates of individual home range obtained from radio-tracking data, observations on Bylot Island, and existing literature (Legagneux et al., 2012; Bilodeau, 2013). We associated the minimum and maximum scores of relative abundance with the minimum and maximum density of individuals, respectively. Ultimately, we calculated the ermine density by linearly interpolating between these two density extremes using the annual index of relative abundance. The index of ermine abundance is derived from observations that include both breeding and potentially non-breeding individuals; consequently, the corresponding estimates of absolute abundance include potential non-breeding individuals.

Arctic fox

We estimated the abundance of Arctic foxes in the study area based on their estimated home range size inside and outside the goose colony. We used the data and methodologies outlined in Dulude-de Broin et al. (2023) to estimate home range size of territorial foxes. However, here, we did not account for annual variations in lemming density as presented in Dulude-de Broin et al. (2023) in order to obtain mean fox home range. Given that foxes are territorial and exhibit an average spatial overlap with adjacent territories of 18% (Clermont et al., 2021), we converted home range size into individual density using the following formula: $density\ of\ individuals = \frac{2}{0.82 \times home\ range}$. We used two as numerator because we assumed each territory was held by a pair of fox, either breeding or non-breeding fox pair, without accounting for potential nomadic or transient individuals. We used values of 12.26 km^2 to represent the mean home range of foxes within the goose colony and 20.02 km^2 for foxes outside the goose colony. We estimated the mean

density of foxes in each zone of the study area according to the mean proportion of the zone covered by the goose colony. We derived the mean annual proportion of each zone covered by the goose colony from the colony outline between 2010 and 2023. We estimated a mean density of 0.14 individuals/km^2 for the study area. Previously, the minimum density of foxes in the study area was estimated to be between 0.03 and 0.13 individuals per km^2 based on camera traps (Royer-Boutin, 2015). Since breeding and non-breeding individuals are both territorial (Lai et al., 2017), the provided estimate of fox abundance includes both breeding and non-breeding individuals.

Table 4: Summary of the lowest, highest, mean and standard deviation of the estimated abundance of each vertebrate species in the vertebrate community of the southern plain of Bylot Island (389 km2). In some cases, two independent approaches have been used to estimate the abundance of the same species as a proxy for uncertainty. We provide a qualitative measure of the method quality based on data available, method used for extrapolation (if necessary), and in some cases, from the fit of statistical models to estimate density. The asterisk (*) indicates that the estimate reflects breeding individuals only. The absence of an asterisk means the abundance estimate may include non-breeding individuals.

Species	Method	Method quality	Justification	Lowest abundance	Highest abundance	Mean abundance	sd	n
Pacific loon	Intensive study area-wide nest monitoring (389 km2)	high	No extrapolation	0*	6*	4*	3	4 (2017-2019, 2022)
Red-throated loon	Intensive study area-wide nest monitoring (389 km2)	high	No extrapolation	42*	76*	64*	15	4 (2017-2019, 2022)
King eider	Intensive, but opportunistic nest monitoring (8 km2) extrapolated by habitat	very low	Intensive, but opportunistic monitoring at relatively small spatial scale, but does not include potential non-breeding individuals			25*		
King eider	Derived from the abundance estimate of red-throated loon using incidental observations	low	Derived from high quality estimate of another species			106		
Long-tailed duck	Intensive, but opportunistic nest monitoring (8 km2) extrapolated by habitat	very low	Intensive, but opportunistic monitoring at relatively small spatial scale, but does not include potential non-breeding individuals			20*		
Long-tailed duck	Derived from the abundance estimate of red-throated loon using incidental observations	low	Derived from high quality estimate of another species			191		
Cackling goose	Extrapolation from intensive nest monitoring (111 km2, R2= 0.75, p = 0.06, n= 5)	high	Strong correlation with study area-wide nest density	8*	87*	34*	26	13 (2004-2016)
Cackling goose	Intensive study area-wide nest monitoring (389 km2)	high	No extrapolation	80*	214*	138*	50	5 (2017-2019, 2022-2023)
Snow goose	Nest monitoring plots extrapolated to mean goose colony area	moderate	Relatively small sample size and uncertainty on goose colony area	2505*	35404*	18129*	11037	11 (1999-2009)
Snow goose	Intensive study area-wide monitoring based on a combination of methods (transects, point counts and nest monitoring plots) and annual colony outline	high	Multiple independent methods and annual colony outline	7982*	47859*	30771*	11962	12 (2010-2019, 2022-2023)
Tundra swan	Intensive study area-wide nest monitoring (389 km2)	high	No extrapolation	0*	2*	1*	1	4 (2017-2019, 2022)
Rough-legged hawk	Extrapolation from intensive nest monitoring (111 km2, R2=0.99, p<0.0001, n=8)	high	Strong correlation with study area-wide nest density	3*	59*	22*	21	6 (2007-2012)
Rough-legged hawk	Intensive study area-wide nest monitoring (389 km2)	high	No extrapolation	0*	66*	30*	29	8 (2013-2019, 2022)

Peregrine falcon	Intensive study area-wide nest monitoring (389 km2)	high	No extrapolation	8*	12*	10*	1	8 (2013-2019, 2022)
Snowy owl	Extrapolation from intensive nest monitoring (111 km2, R2=0.98, p<0.0001, n=10)	high	Strong correlation with study area-wide nest density	0*	65*	14*	23	16 (1996-2011)
Snowy owl	Intensive study area-wide nest monitoring (389 km2)	high	No extrapolation	0*	144*	17*	45	10 (2012-2019, 2022-2023)
Rock ptarmigan	Intensive, but opportunistic nest monitoring (8 km2) extrapolated to study area	very low	Intensive, but opportunistic monitoring at relatively small spatial scale and prime nesting habitat not well sampled			24*		
Sandhill crane	Intensive nest monitoring (33 km2) extrapolated to study area	moderate	Nest density is extrapolated uniformly across the study area			69*		
American golden-plover	Distance sampling throughout lowland (313 km2)	high	Large sample size	397*	1725*	1102*	432	8 (2014-2019, 2022-2023)
Black-bellied plover	Derived from the abundance estimate of American golden-plover using transects observations	low	Derived from high quality estimate of another species			29*		
Black-bellied plover	Derived from the abundance estimate of American golden-plover using incidental observations	very low	Derived from high quality estimate of another species, but potentially includes transient migratory individuals			87		
Common-ringed plover	Nest monitoring on the main breeding sites	moderate	Intensive monitoring, but not exhaustive to study area	44*	62*	55*	9	3 (2015-2017)
Ruddy turnstone	Derived from the abundance estimate of Bairds sandpiper using transects observations	low	Derived from moderate quality estimate of another species			36*		
Ruddy turnstone	Derived from the abundance estimate of Bairds sandpiper using incidental observations	very low	Derived from moderate quality estimate of another species, but potentially includes transient migratory individuals			47		
Red knot	Derived from the abundance estimate of Bairds sandpiper using transects observations	low	Derived from moderate quality estimate of another species			59*		
Red knot	Derived from the abundance estimate of Bairds sandpiper using incidental observations	very low	Derived from moderate quality estimate of another species, but potentially includes transient migratory individuals			206		
Pectoral sandpiper	Derived from the abundance estimate of Bairds sandpiper using transects observations	low	Derived from moderate quality estimate of another species			71*		

Pectoral sandpiper	Derived from the abundance estimate of Bairds sandpiper using incidental observations	very low	Derived from moderate quality estimate of another species, but potentially includes transient migratory individuals			226		
Baird's sandpiper	Extrapolation from intensive nest monitoring (2 km2) and transects observations	moderate	Uncertain relation between nest density and transects observations			2170*		
White-rumped sandpiper	Derived from the abundance estimate of Bairds sandpiper using transects observations	low	Derived from moderate quality estimate of another species			878*		
White-rumped sandpiper	Derived from the abundance estimate of Bairds sandpiper using incidental observations	very low	Derived from moderate quality estimate of another species, but potentially includes transient migratory individuals			1005		
Buff-breasted sandpiper	Derived from the abundance estimate of Bairds sandpiper using transects observations	low	Derived from moderate quality estimate of another species			5*		
Buff-breasted sandpiper	Derived from the abundance estimate of Bairds sandpiper using incidental observations	very low	Derived from moderate quality estimate of another species, but potentially includes transient migratory individuals			7		
Red phalarope	Derived from the abundance estimate of Bairds sandpiper using transects observations	low	Derived from moderate quality estimate of another species			124*		
Red phalarope	Derived from the abundance estimate of Bairds sandpiper using incidental observations	very low	Derived from moderate quality estimate of another species, but potentially includes transient migratory individuals			240		
Glaucous gull	Extrapolation from intensive nest monitoring (111 km2, R2=0.7, p=0.16, n=4)	high	Strong correlation with study area-wide nest density	59*	80*	73*	6	13 (2004-2016)
Glaucous gull	Intensive study area-wide nest monitoring (389 km2)	high	No extrapolation	60*	80*	71*	9	4 (2017-2019, 2022)
Long-tailed jaeger	Intensive nest monitoring (33 km2) extrapolated by habitat	high	Relatively large spatial coverage of sampling	0*	900*	272*	285	17 (2004-2019, 2022)
Parasitic jaeger	Maximum number of individuals banded in a year	low	Based on a single year and potentially not all individuals were captured			17		
Parasitic jaeger	Maximum number of nest found annually during study area-wide opportunistic nest monitoring	very low	Monitoring does not include potential non-breeding individuals			8*		
Common raven	Derived from the abundance estimate of glaucous gull using transects observations	very low	Derived from moderate quality estimate of another species, but potential difference in detectability between species			14		

Common raven	Derived from the abundance estimate of glaucous gull using incidental observations	very low	Derived from moderate quality estimate of another species, but potential difference in detectability between species			18		
Horned lark	Derived from the abundance estimate of Lapland longspur using transects observations	low	Derived from moderate quality estimate of another species			310*		
Horned lark	Derived from the abundance estimate of Lapland longspur using incidental observations	low	Derived from moderate quality estimate of another species			352*		
American pipit	Derived from the abundance estimate of Lapland longspur using transects observations	very low	Derived from moderate quality estimate of another species and prime nesting habitat not sampled			46*		
American pipit	Derived from the abundance estimate of Lapland longspur using incidental observations	low	Derived from moderate quality estimate of another species and prime nesting habitat not well sampled			74*		
Lapland longspur	Extrapolation from intensive nest monitoring (2 km2) and transects observations	moderate	Uncertain relation between nest density and transects observations			6080*		
Snow bunting	Derived from the abundance estimate of Lapland longspur using transects observations	very low	Derived from moderate quality estimate of another species and prime nesting habitat not sampled			15*		
Snow bunting	Derived from the abundance estimate of Lapland longspur using incidental observations	low	Derived from moderate quality estimate of another species and prime nesting habitat not well sampled			236*		
Nearctic brown lemming	Rigorous density estimates at small spatial scale (0.22 km2) extrapolated by habitat	moderate	Intensive sampling, but small spatial coverage and extrapolation by habitat	724	447630	54170	93455	27 (1995-2019, 2021-2022)
Nearctic collared lemming	Rigorous density estimates at small spatial scale (0.22 km2) extrapolated by habitat	moderate	Intensive sampling, but small spatial coverage and extrapolation by habitat	724	39302	8306	10202	27 (1995-2019, 2021-2022)
Arctic hare	Derived from the abundance estimate of Arctic fox using incidental observations	very low	Derived from moderate quality estimate of another species, prime nesting habitat not well sampled and difference in detectability between species			6		
American ermine	Indices of relative abundance derived from testimonials converted to abundance using home range size	moderate	Indirect indices and uncertainty on ermine home range size estimates	8	156	40	37	27 (1993-2019)
Arctic fox	Derived from extensive fox home range size studies (n=109)	moderate	Indirect indices, but large sample size			53		

Figure 11: Time series of the estimated annual abundance of vertebrate species on the southern plain of Bylot Island (389 km^2). Estimated abundance represents adult individuals, with the exception of lemmings, for which juveniles were also included in the estimate. Time series shorter than 5 years are not presented.

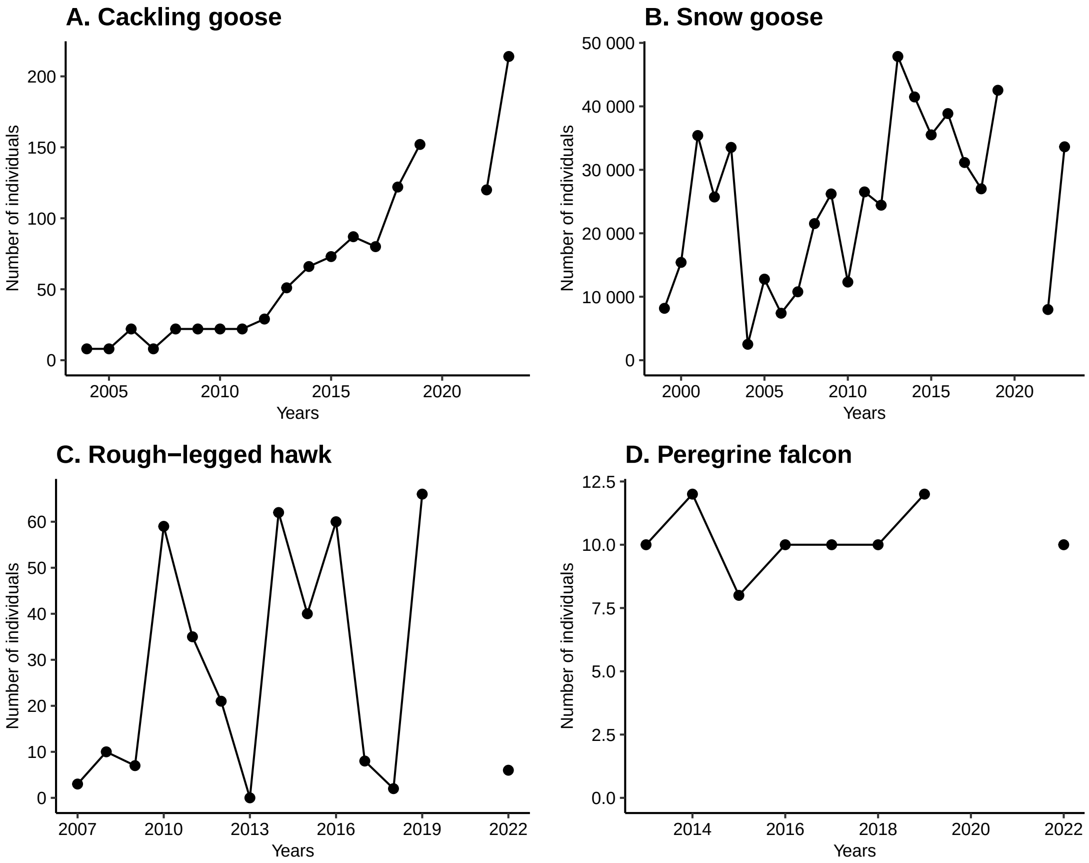

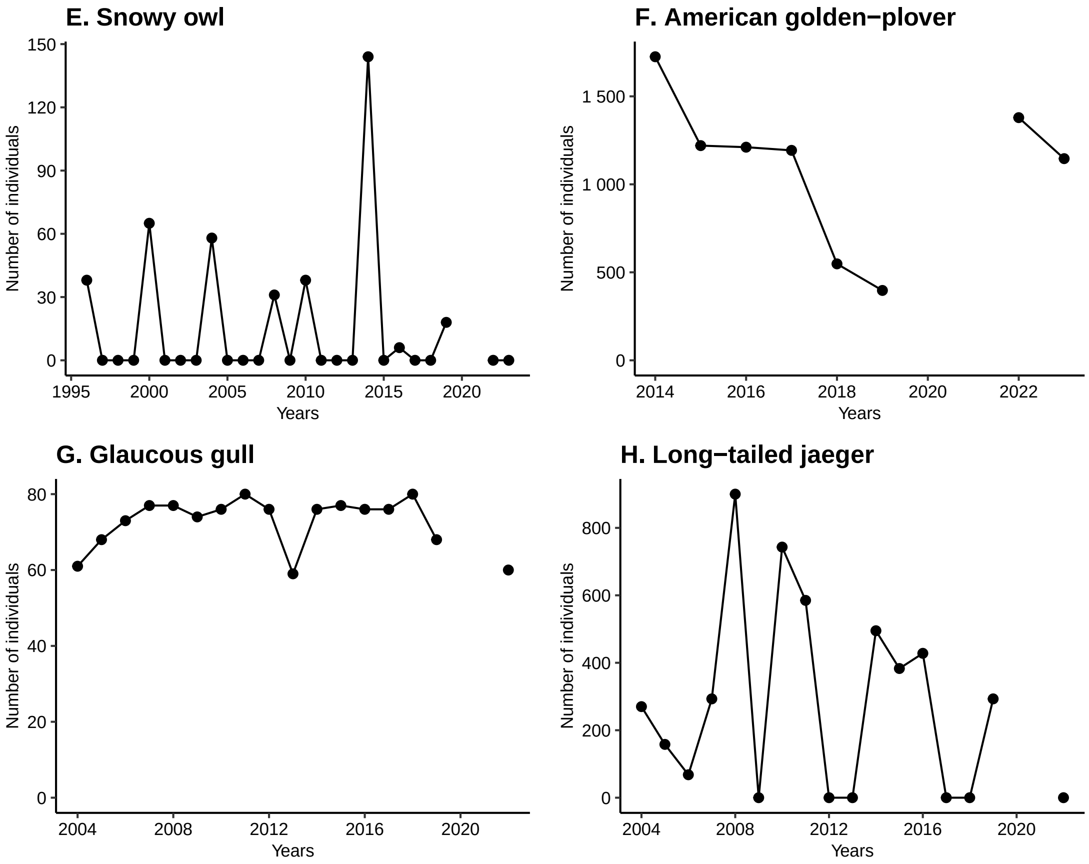
E. Snowy owl
Number of individuals
0
30
60
90
120
150
1995
2000
2005
2010
2015
2020
Years
F. American golden−plover
Number of individuals
0
500
1 000
1 500
2014
2016
2018
2020
2022
Years
G. Glaucous gull
Number of individuals
0
20
40
60
80
2004
2008
2012
2016
2020
Years
H. Long−tailed jaeger
Number of individuals
0
200
400
600
800
2004
2008
2012
2016
2020
Years

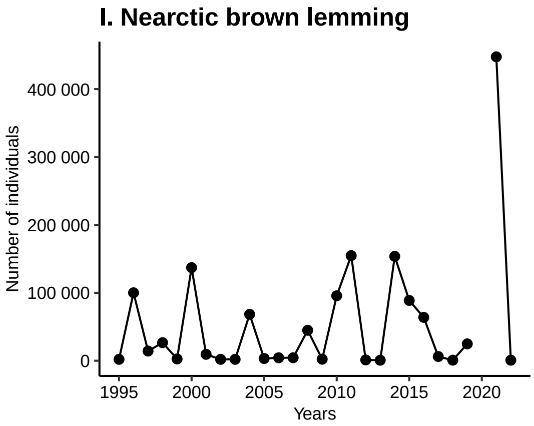
I. Nearctic brown lemming
Number of individuals
400 000
300 000
200 000
100 000
0
1995
2000
2005
2010
2015
2020
Years

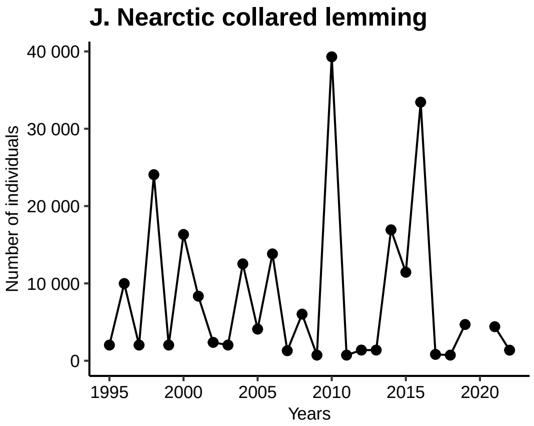
J. Nearctic collared lemming
Number of individuals
40 000
30 000
20 000
10 000
0
1995
2000
2005
2010
2015
2020
Years

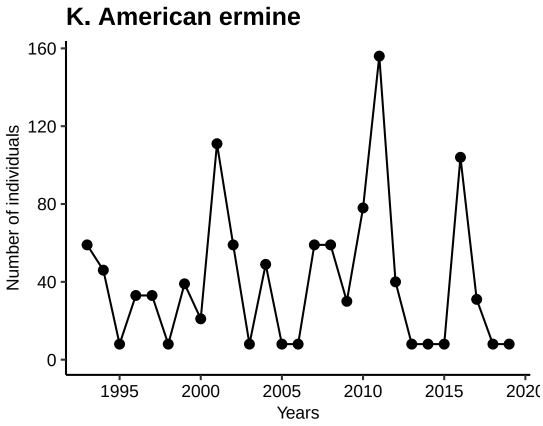
K. American ermine
Number of individuals
160
120
80
40
0
1995
2000
2005
2010
2015
Years

Table 5: Due to the absence of confidence intervals in our abundance estimates, we present uncertainty intervals based on field expert judgment. Experts derived these intervals by considering the given abundance estimate, estimates for other species for comparaison, and their field expertise. For species with time series data (several years of estimates), the intervals are presented for the lowest and highest abundance reached within the given time series. These intervals reflect the interval within which the actual abundance lies according to field experts. For species without time series, the intervals are presented for the mean only.

		Annual abundance (individuals)		
Species	Period	Lowest	Highest	Mean
Pacific loon	2017-2019, 2022	[0-6]	[6-10]	
Red-throated loon	2017-2019, 2022	[42-62]	[75-100]	
King eider	Mean abundance			[60-250]
Long-tailed duck	Mean abundance			[80-300]
Cackling goose	2004-2016	[8-30]	[87-110]	
Cackling goose	2017-2019, 2022-2023	[80-110]	[214-244]	
Snow goose	1999-2009	[2500-10000]	[35000-60000]	
Snow goose	2010-2019, 2022-2023	[6000-10000]	[45000-60000]	
Tundra swan	2017-2019, 2022	[0-2]	[2-6]	
Rough-legged hawk	2007-2012	[0-8]	[50-90]	
Rough-legged hawk	2013-2019, 2022	[0-4]	[66-86]	
Peregrine falcon	2013-2019, 2022	[8-12]	[12-20]	
Snowy owl	1996-2011	0	[50-100]	
Snowy owl	2012-2019, 2022-2023	0	[144-170]	
Rock ptarmigan	Mean abundance			[10-60]
Sandhill crane	Mean abundance			[15-45]
American golden-plover	2014-2019, 2022-2023	[300-600]	[1000-2500]	
Black-bellied plover	Mean abundance			[6-30]
Common-ringed plover	2015-2017	[44-60]	[60-100]	
Ruddy turnstone	Mean abundance			[10-30]
Red knot	Mean abundance			[10-30]
Pectoral sandpiper	Mean abundance			[20-100]
Baird's sandpiper	Mean abundance			[1500-3500]
White-rumped sandpiper	Mean abundance			[1000-2000]
Buff-breasted sandpiper	Mean abundance			[2-10]
Red phalarope	Mean abundance			[20-80]
Glaucous gull	2004-2016	[50-80]	[70-100]	
Glaucous gull	2017-2019, 2022	[60-80]	[80-100]	
Long-tailed jaeger	2004-2019, 2022	[0-10]	[300-900]	
Parasitic jaeger	Mean abundance			[15-50]
Common raven	Mean abundance			[30-75]
Horned lark	Mean abundance			[200-600]
American pipit	Mean abundance			[50-300]
Lapland longspur	Mean abundance			[6000-10000]
Snow bunting	Mean abundance			[50-300]
Nearctic brown lemming	1995-2019, 2021-2022	[100-2000]	[200000-450000]	
Nearctic collared lemming	1995-2019, 2021-2022	[100-2000]	[20000-50000]	
Arctic hare	Mean abundance			[15-50]
American ermine	1993-2019	[0-10]	[50-200]	
Arctic fox	Mean abundance			[30-60]

b. Taxonomy and systematics

Birds taxonomy was obtained from the IOC World Bird List 14.2 (Gill et al., 2024) and mammals taxonomy from the Mammal species of the world: a taxonomic and geographic reference (Upham et al., 2024).

c. Permit history

All research involving animals on Bylot Island has been approved by an institutional Animal Care Committee. In 1999, the study area became part of Sirmilik National Park, managed by Parks Canada. Since then, all research activities in the park have been approved by a Joint Park Management Committee.

d. Project personnel

Principal and associated investigators

Gilles Gauthier, Austin Reed, Jean-François Giroux, Dominique Berteaux, Joël Bêty, Josée Lefebvre, Dominique Gravel, Jean-François Therrien, Nicolas Lecomte, Dominique Fauteux, Pierre Legagneux (see Gauthier et al. 2024a)

Students

By combining animal and plant ecology, 24 doctoral theses and 56 master theses have been completed in relation to the study area located on the south plain of Bylot Island (see Gauthier et al. 2024a).

Class III. Data set status and accessibility

A. Status

1. Latest update

August 11, 2025

2. Latest archive date

August 11, 2025

3. Metadata status

August 11, 2025

4. Data verification

The methods employed to estimate species abundance were subject to several rounds of revision by the authors.

B. Accessibility

1. Storage location and medium

The complete data set is available as Supporting Information. The complete data set, including raw data, is also archived in Dryad: `https://doi.org/10.5061/dryad.44j0zpcnt`. The code and the complete R project used to estimate species abundance are archived in Zenodo: https://doi.org/10.5281/zenodo.16794619.

2. Contact persons

Overall project

Joël Bêty; *joel_bety@uqar.ca*; 418 723-1986 #1701; 300 allée des Ursulines, Rimouski, Québec, Canada, G5L 3A1, Office B-002

Specific subproject description

Louis Moisan, *louis.moisan.bio@gmail.com*

3. Copyright restrictions

None

4. Proprietary restrictions

a. Release date

None

b. Citation

Please refer to the data paper version published in *Ecology* (Moisan et al., 2025).

c. Disclaimer

None

5. Costs

None, the data can be used free of charge.

Class IV. Data structural descriptors

A. Data set file

1. Identity

a. BYLOT-species_taxonomy.csv
b. BYLOT-species_density_monitoring.csv
c. BYLOT-species_abundance.csv
d. BYLOT-community_composition.csv
e. BYLOT-species_body_mass.csv

2. Size

a. 35 records, not including header row (4.1 kB)
b. 1186 records, not including header row (128.5 kB)
c. 261 records, not including header row (35.6 kB)
d. 35 records, not including header row (4.3 kB)
e. 54 records, not including header row (3.8 kB)

3. Format and storage mode

All files are in a comma-separated value format (.csv).

4. Header information

a. BYLOT-species_taxonomy.csv

class; order; family; genus; species_scientific; species_en; species_fr; functional_group; migratory_status

b. BYLOT-species_density_monitoring.csv

species_en; year; zone; area_sampled_km2; habitat; method; monitoring; breeding_status; ind_density_km2

c. BYLOT-species_abundance.csv

species_en; year; breeding_status; abundance; method_description; method_quality; spatial_extrapolation

d. BYLOT-community_composition.csv

species_en; 1993 ... 2023; mean

e. BYLOT-species_body_mass.csv

species_en; site;mean_body_mass_g; sample_size; reference

5. Alphanumeric attributes

Mixed

6. Special characters/fields

Unavailable values are indicated by NA.

7. Authentication procedures

Sums of the numeric columns:
b. BYLOT-species_density_monitoring.csv: year= 2389178; area_sampled_km2= 62017.96; ind_density_km2= 31269.65
c. BYLOT-species_abundance.csv: year= 456443; abundance= 2286541
d. BYLOT-community_composition.csv: 1993= 59; 1994= 46; 1995= 4060; 1996= 110133; 1997= 16284; 1998= 50661; 1999= 12880; 2000= 168935; 2001= 53306; 2002= 30172; 2003= 37592; 2004= 83856; 2005= 20380; 2006= 25726; 2007= 16886; 2008= 73475; 2009= 29315; 2010= 148246; 2011= 182925; 2012= 27161; 2013= 50088; 2014= 214747; 2015= 137586; 2016= 138211; 2017= 39625; 2018= 29379; 2019= 73157; 2021= 452032; 2022= 11778; 2023= 34986; mean= 10575
e. BYLOT-species_body_mass.csv: body_mass_g= 51256; sample_size= 14138

B. Variable information

1. Variable identity

See Table 6

2. Variable definition

See Table 6

3. Units of measurement

See Table 6

Table 6: Summary of variable definition and unit of measurement.

Data file	Variable identity	Variable definition	Units
a.	class	Taxonomic class for birds (Gill et al., 2024) and mammals species (Upham et al., 2024).	NA
a.	order	Taxonomic order for birds (Gill et al., 2024) and mammals species (Upham et al., 2024).	NA
a.	family	Taxonomic family for birds (Gill et al., 2024) and mammals species (Upham et al., 2024).	NA
a.	genus	Taxonomic genus for birds (Gill et al., 2024) and mammals species (Upham et al., 2024).	NA
a.	species_scientific	Taxonomic species for birds (Gill et al., 2024) and mammals species (Upham et al., 2024).	NA
a.	species_en	Common names of species in English.	NA
a.	species_fr	Common names of species in French.	NA
a.	functional_group	Functional group for each species. The classification of species into functional groups is based on Moisan et al. (2023).	NA
a.	migratory_status	Migratory status of each species. The classification of species migratory status is based on Gauthier et al., (2011) and Moisan et al. (2023).	NA
b.	species_en	Common names of species in English.	NA
b.	year	Year in which species density was monitored in the corresponding zone.	years
b.	zone	Zone of the study area in which density was measured.	NA
b.	area_sampled_km2	Area (in square kilometers) sampled to measure species density.	square kilometers
b.	habitat	Habitat in which species density was measured.	NA
b.	method	Method used to measure species density.	NA
b.	monitoring	Type of monitoring conducted, distinguishing between systematic (planned, standardized data collection) and opportunistic (irregular or incidental).	NA
b.	breeding_status	Reproductive status of the individuals.	NA
b.	ind_density_km2	Density of individuals (number of individuals per square kilometer) measured in the corresponding year and zone of the study area.	individuals per square kilometer
c.	species_en	Common names of species in English.	NA
c.	year	Year corresponding to the estimate of annual abundance. If abundance has not been calculated for a given series of years, but rather as a general average, then NA has been assigned.	years
c.	breeding_status	Reproductive status of the individuals.	NA
c.	abundance	Estimate of the annual number of individuals found within the 389 km2 study area located on the southern part of Bylot Island during the breeding season (May to August). The estimates only consider adults, with the exception of lemmings, for which no distinction has been made between juveniles and adults.	individuals
c.	method_description	Brief overview of the method used to estimate the species abundance.	NA
c.	method_quality	Qualitative measure of the method quality based on data available, method used for extrapolation (if necessary), and in some cases, from the fit of statistical models to estimate density.	NA

c.	spatial_extrapolation	Indicates whether the abundance estimate was derived from monitoring across the entire study area ("no") or was spatially extrapolated ("yes").	NA
d.	species_en	Common names of species in English.	NA
d.	1993 – 2023	Estimate of the annual number of individuals within the 389 km2 study area located on the southern part of Bylot Island during the breeding season (May to August) for the corresponding year. The estimates represent only breeding individuals, except for lemmings, for which the estimates include both breeding and non-breeding individuals. The estimates exclude non-breeding individuals that stop for only a few days during their migration. The estimates only consider adults, with the exception of lemmings, for which no distinction has been made between juveniles and adults. Note that 2020 does not appear since fieldwork was not possible due to Covid-19 restrictions.	individuals
d.	mean	Estimate of the long-term average annual number of individuals within the 389 km2 study area located on the southern part of Bylot Island during the breeding season (May to August). This includes both breeding and non-breeding individuals that stay in the study area for a significant period of time, and excludes non-breeding individuals that stop for only a few days during their migration. The estimates only consider adults. For species where abundance was estimated using multiple methods, we selected the method determined to be of the highest quality. In cases where multiple methods yielded equivalent quality, the mean value of their estimates was retained.	individuals
e.	species_en	Common names of species in English.	NA
e.	site	Site where body mass measurements were taken.	NA
e.	mean_body_mass_g	Mean individual body mass.	grams
e.	sample_size	Number of individuals measured.	individuals
e.	reference	Reference from which estimate of mean body mass were derived.	NA

4. Data type

a. Storage type

See Table 7

b. List and definition of variable codes

See Table 7

c. Range for numeric values

See Table 7

d. Missing value codes

Unavailable values are indicated by NA.

e. Number of digits

See Table 7

Table 7: Summary of variable storage type, code definition, range and number of digit.

Data file	Variable identity	Storage type	Definition variable codes	Range	Number digits
a.	class	string	NA	NA	NA
a.	order	string	NA	NA	NA
a.	family	string	NA	NA	NA
a.	genus	string	NA	NA	NA
a.	species_scientific	string	NA	NA	NA
a.	species_en	string	NA	NA	NA
a.	species_fr	string	NA	NA	NA
a.	functional_group	string	NA	NA	NA
a.	migratory_status	string	resident: Individuals performing movements within the study area throughout the annual cycle. \ partial migrant: A combination of resident and migratory and/or individuals performing long-distance foraging trips outside the study area during the non-breeding period. \ migrant: Individuals performing seasonal and highly synchronous movements between the study area and a distant non-breeding ground.	NA	NA
b.	species_en	string	NA	NA	NA
b.	year	integer	NA	1995-2023	0
b.	zone	string	see Figure 4 and 5 in MetadataS1.pdf or file study_area/study_area.shp for spatial delineation of the zones.	NA	NA
b.	area_sampled_km2	numeric	NA	0.11-388.80	2
b.	habitat	string	see section c. Habitat for a detailed description of mesic, wetland and upland habitats. mixed is used to represent a combination of different habitats.	NA	NA
b.	method	string	nest sampling: Total number of nests found within a given zone or plot. / trapping: Capture-mark-recapture of individuals within a trapping grid to estimate density. / distance sampling: Observations of individuals along vertebrate count transects analyzed using a distance detection function to estimate density.	NA	NA
b.	monitoring	string	opportunistic: irregular or incidental data collection. \ systematic: planned and standardized data collection.	NA	NA
b.	breeding_status	string	breeding: Individuals present in the study area during the breeding period (June to August) and having attempted and/or completed breeding. \ undetermined: Individuals present on the study area during the breeding period (June to August) that might have breed or not.	NA	NA
b.	ind_density_km2	numeric	NA	0.000-1550.000	3
c.	species_en	string	NA	NA	NA
c.	year	integer	If abundance has not been calculated for a given series of years, but rather as a general average, then NA has been assigned.	1993-2023	0

c.	breeding_status	string	breeding: Individuals present in the study area during the breeding period (June to August) and having attempted and/or completed breeding. \ undetermined: Individuals present on the study area during the breeding period (June to August) that might have breed or not.	NA	NA
c.	abundance	integer	NA	0-447630	0
c.	method_description	string	NA	NA	NA
c.	method_quality	string	very low: Sampling might not encompasses prime nesting habitat, excludes transient migratory individuals or includes potential non-breeding individuals. If abundance is derived from the abundance estimate of another species based relative abundance, detection probabilities may differ. \ low: Abundance is derived from the estimate of another species based on indices of relative abundance. \ moderate: Small to intermediate scale sampling with spatial extrapolation. \ high: Large scale intensive sampling, with some spatial extrapolation in a few cases.	NA	NA
c.	spatial_extrapolation	binary	yes: Some form of spatial extrapolation has been applied to estimate species abundance at the landscape scale. / no: Monitoring was performed at the landscape scale without requiring spatial extrapolation.	NA	NA
d.	species_en	string	NA	NA	NA
d.	1993	integer	NA	59-59	0
d.	1994	integer	NA	46-46	0
d.	1995	integer	NA	8-2026	0
d.	1996	integer	NA	33-100080	0
d.	1997	integer	NA	0-14225	0
d.	1998	integer	NA	0-26590	0
d.	1999	integer	NA	0-8175	0
d.	2000	integer	NA	21-137110	0
d.	2001	integer	NA	0-35404	0
d.	2002	integer	NA	0-25715	0
d.	2003	integer	NA	0-33532	0
d.	2004	integer	NA	8-68378	0
d.	2005	integer	NA	0-12773	0
d.	2006	integer	NA	0-13817	0

d.	2007	integer	NA	0-10778	0
d.	2008	integer	NA	10-44827	0
d.	2009	integer	NA	0-26199	0
d.	2010	integer	NA	22-95615	0
d.	2011	integer	NA	0-154805	0
d.	2012	integer	NA	0-24424	0
d.	2013	integer	NA	0-47859	0
d.	2014	integer	NA	8-153753	0
d.	2015	integer	NA	0-88772	0
d.	2016	integer	NA	6-63865	0
d.	2017	integer	NA	0-31147	0
d.	2018	integer	NA	0-27001	0
d.	2019	integer	NA	2-42535	0
d.	2021	integer	NA	4402-447630	0
d.	2022	integer	NA	0-7982	0
d.	2023	integer	NA	0-33626	0
d.	mean	integer	NA	5-6080	0
e.	species_en	string	NA	NA	NA
e.	site	string	bylot: Southern plain of Bylot Island, Nunavut, Canada.\ baffin: Baffin Island (65.421 N, 70.966 E), Nunavut, Canada. \ undetermined: Data were not retrieved from original publications.	NA	NA
e.	mean_body_mass_g	integer	NA	21-6378	0
e.	sample_size	integer	NA	1-6405	0
e.	reference	string	NA	NA	NA
e.	species_en	string	NA	NA	NA

C. Data anomalies: Description of missing data, anomalous data, calibration errors, etc.

c. BYLOT-species_abundance.csv

`year`: If abundance has not been calculated for a given series of years, but rather as a general average, then NA has been assigned.

d. BYLOT-community_composition.csv

`1993 - 2023`: For the years 1993 to 2023, NA values in the respective columns indicate that no annual abundance estimates were available for the study area.
`mean`: The 'mean' column displays values only when annual estimates (i.e., time series data) were unavailable.

e. BYLOT-species_body_mass.csv

`sample_size`: NA is used when sample size was not specified.

Class V. Supplemental descriptors

A. Data acquisition

1. Data forms or acquisition methods

See Section **2. Experimental or sampling design**

2. Location of completed data forms

The complete data set is available as Supporting Information. The complete data set, including raw data, is also archived in Dryad: `https://doi.org/10.5061/dryad.44j0zpcnt`. Also, part of the raw monitoring data for the key species of the food web are also available at the NordicanaD data repository (`https://nordicana.cen.ulaval.ca/en/list-of-publications.php`; Lemming monitoring on Bylot Island; Monitoring of Greater Snow Goose reproduction on Bylot Island; Monitoring of Lapland longspur reproduction on Bylot Island; Monitoring of shorebirds reproduction on Bylot Island; Monitoring of arctic and red fox reproduction on Bylot Island; Monitoring of avian predator reproduction on Bylot Island; Relative abundance of tundra bird and mammal species encountered daily on Bylot Island) and are periodically updated as the field studies continue on Bylot Island.

3. Data entry verification procedures

The methods used to extract final species abundance estimates were subject to several rounds of revision by the authors.

B. Quality assurance/quality control procedures

The authors revised the presented measures of species density and abundance estimates. Following comments from two anonymous reviewers in April 2025, the methods, dataset, and MetadataS1 document were updated accordingly.

C. Computer programs and data-processing algorithms

1. Program

R version 4.4.1 (2024-06-14)

2. Operating system

Data preparation was performed on x86_64-pc-linux-gnu (64-bit) with Ubuntu 22.04.3 LTS.

3. Packages

dplyr 1.1.4 (Wickham et al., 2023a)
tidyr 1.3.1 (Wickham et al., 2024)
sf 1.0-20 (Pebesma et al., 2018)
stringr 1.5.1 (Wickham, 2023)
xtable 1.8-4 (Dahl et al., 2019)
Distance 1.0.9 (Miller et al., 2019)
ggplot2 3.5.1 (Wickham, 2016)
lme4 1.1-35.3 (Bates et al., 2015)
AICcmodavg 2.3-3 (Mazerolle, 2023)
scales 1.3.0 (Wickham et al., 2023b)
ggmap 4.0.1 (Kahle and Wickham, 2013)
ggspatial 1.1.9 (Dunnington, 2023)

4. Code

The code and the complete R project used to estimate species abundance are archived in Zenodo: https://doi.org/10.5281/zenodo.16794619.

D. Archiving

1. Archival procedures

The complete data set is available as Supporting Information. The complete data set, including raw data, is also archived in Dryad: `https://doi.org/10.5061/dryad.44j0zpcnt`. The code and the complete R project used to estimate species abundance are archived in Zenodo: https://doi.org/10.5281/zenodo.16794619.

2. Redundant archival sites

The complete data set is available as Supporting Information, on Dryad `https://doi.org/10.5061/dryad.44j0zpcnt` and within the complete R project on Zenodo https://doi.org/10.5281/zenodo.16794619.

E. Publications and results

The presented estimates of species abundance and body mass are used in:
Moisan, L., D. Gravel, G. Gauthier, P. Legagneux and J. Bêty, 2025. Arctic migrations shape global meta-communities: contrasting insights from species occurrence, abundance and biomass. *Global Ecology And Biogeography.* `https://doi.org/10.1111/geb.70074`

Previous estimates of species abundance on the southern plain of Bylot Island were presented by Legagneux et al. (2012), however, the temporal series presented here is longer, the methods are refined and the taxonomic resolution is higher.

F. History of data set usage

1.Data request history

None

2.Data set update history

The first version of the dataset was archived on Dryad prior to submission to a peer-reviewed journal on November 19, 2024. Following comments from anonymous reviewers, a revised version of the dataset was archived on June 23 2025. Minor details were corrected on August 11 2025 following a final review by the subject-matter editor, the authors and the editorial staff. On August 12, 2025, the hyperlink to the complete R project, which previously redirected to version 1 of the repository (https: // zenodo.org/records/13993827), was updated to point to the general software repos-itory containing the up-to-date version (https://doi.org/10.5281/zenodo.16794619).

3.Review history

Following comments from two anonymous reviewers in April 2025, the methods, dataset, and MetadataS1 document were updated accordingly. Minor details were corrected on August 11 2025 following a final review by the subject-matter editor, the authors and the editorial staff.

4.Questions and comments from secondary users

None

Acknowledgements

We are deeply appreciative of the extensive data gathered over the decades by generations of students and researchers during their fieldwork at the Bylot Island research station, which made this project possible. Our gratitude also extends to the Center of Northern Studies for providing research facilities, as well as to the Polar Continental Shelf Program and Sirmilik National Park (Parks Canada) for their logistical support throughout the years. Additionally, we would like to express our special thanks to the Mittimatalik community and the Mittimatalik Hunters and Trappers Organization for their ongoing support of ecological monitoring on Bylot Island and for permitting us to conduct research on their land.

References

Andersson, M. (1971). Breeding behaviour of the long-tailed skua stercorarius longicaudus (vieillot). *Ornis Scandinavica*, 2(1):35–54.

Bates, D., Mächler, M., Bolker, B., and Walker, S. (2015). Fitting linear mixed-effects models using lme4. *Journal of Statistical Software*, 67(1):1–48.

Beardsell, A., Gauthier, G., Therrien, J.-F., and Bêty, J. (2016). Nest site characteristics, patterns of nest reuse, and reproductive output in an arctic-nesting raptor, the rough-legged hawk. *The Auk: Ornithological Advances*, 133(4):718–732.

Bilodeau, F. (2013). Effet du couvert nival, de la nourriture et de la prédation hivernale sur la dynamique de population des lemmings. Master's thesis, Université Laval, Québec, Québec.

Bolduc, D., Fauteux, D., Gagnon, C. A., Gauthier, G., Bêty, J., and Legagneux, P. (2023). Testimonials to reconstruct past abundances of wildlife populations. *Basic and Applied Ecology*, 68:23–34.

Bolduc, D., Fauteux, D., Gauthier, G., and Legagneux, P. (2025). Seasonal role of a specialist predator in rodent cycles: Ermine–lemming interactions in the high arctic. *Ecology*, 106(1):e4512.

Brown, J. H. and Heske, E. J. (1990). Temporal changes in a chihuahuan desert rodent community. *Oikos*, 59(3):290–302.

Centre for Northern Studies (2024). Climate station data from bylot island in nunavut, canada, v. 1.13.0 (1992-2023). Nordicana D2, doi: 10.5885/45039SL-EE76C1BDAADC4890.

Centre of Northern Studies and Laval University (2019). Long-term climate observations on bylot island. `https://bylot.cen.ulaval.ca/en/climatetrend.php`. Accessed: 2024-10-03.

Christin, S., St-Laurent, M.-H., and Berteaux, D. (2015). Evaluation of argos telemetry accuracy in the high-arctic and implications for the estimation of home-range size. *PLoS One*, 10(11):e0141999.

Clermont, J., Grenier-Potvin, A., Duchesne, É., Couchoux, C., Dulude-de Broin, F., Beardsell, A., Bêty, J., and Berteaux, D. (2021). The predator activity landscape predicts the anti-predator behavior and distribution of prey in a tundra community.

Ecosphere, 12(12):e03858.

Cohen, J. E., Jonsson, T., and Carpenter, S. R. (2003). Ecological community description using the food web, species abundance, and body size. *Proceedings of the national academy of sciences*, 100(4):1781–1786.

Corbeil-Robitaille, M.-Z., Duchesne, É., Fortier, D., Kinnard, C., and Bêty, J. (2024). Linking geomorphological processes and wildlife microhabitat selection: nesting birds select refuges generated by permafrost degradation in the arctic. *Biogeosciences*, 21(14):3401–3423.

Dahl, D. B., Scott, D., Roosen, C., Magnusson, A., and Swinton, J. (2019). *xtable: Export Tables to LaTeX or HTML*. R package version 1.8-4.

Dahood, A., de Mutsert, K., and Watters, G. M. (2020). Evaluating antarctic marine protected area scenarios using a dynamic food web model. *Biological conservation*, 251:108766.

David, P., Thebault, E., Anneville, O., Duyck, P.-F., Chapuis, E., and Loeuille, N. (2017). Impacts of invasive species on food webs: a review of empirical data. *Advances in ecological research*, 56:1–60.

Duchesne, É., Lamarre, J.-F., Gauthier, G., Berteaux, D., Gravel, D., and Bêty, J. (2021). Variable strength of predator-mediated effects on species occurrence in an arctic terrestrial vertebrate community. *Ecography*, 44(8):1236–1248.

Dulude-de Broin, F., Clermont, J., Beardsell, A., Ouellet, L.-P., Legagneux, P., Bêty, J., and Berteaux, D. (2023). Predator home range size mediates indirect interactions between prey species in an arctic vertebrate community. *Journal of Animal Ecology*, 92(12):2373–2385.

Dunnington, D. (2023). *ggspatial: Spatial Data Framework for ggplot2*. R package version 1.1.9.

Fauteux, D., , G., Mazerolle, M. J., Coallier, N., Bêty, J., and Berteaux, D. (2018). Evaluation of invasive and non-invasive methods to monitor rodent abundance in the arctic. *Ecosphere*, 9(2):e02124.

Fauteux, D., Gauthier, G., and Berteaux, D. (2015). Seasonal demography of a cyclic lemming population in the canadian arctic. *Journal of Animal Ecology*, 84(5):1412–1422.

Gauthier, G. (2020). Lemming monitoring on bylot island, nunavut, canada.

https://nordicana.cen.ulaval.ca/dpage.aspx?doi=45400AW-9891BD76704C4CE2.

Gauthier, G., Berteaux, D., Bêty, J., Legagneux, P., Fauteux, D., Gravel, D., and Cadieux, M.-C. (2024a). Scientific contributions and lessons learned from 30 years of ecological monitoring of the bylot island tundra ecosystem. *Frontiers in Ecology and Evolution*, 12:1359745.

Gauthier, G., Berteaux, D., Bêty, J., Tarroux, A., Therrien, J.-F., McKinnon, L., Legagneux, P., and Cadieux, M.-C. (2011). The tundra food web of bylot island in a changing climate and the role of exchanges between ecosystems. *Ecoscience*, 18(3):223–235.

Gauthier, G., Bêty, J., Cadieux, M.-C., Legagneux, P., Doiron, M., Chevallier, C., Lai, S., Tarroux, A., and Berteaux, D. (2013). Long-term monitoring at multiple trophic levels suggests heterogeneity in responses to climate change in the canadian arctic tundra. *Philosophical Transactions of the Royal Society B: Biological Sciences*, 368(1624):20120482.

Gauthier, G. and Cadieux, M. (2020a). Monitoring of greater snow goose reproduction on bylot island, nunavut, canada. https://nordicana.cen.ulaval.ca/dpage.aspx?doi=45570CE-2D00DCA728074FA7.

Gauthier, G. and Cadieux, M. (2020b). Relative abundance of tundra bird and mammal species encountered daily on bylot island, nunavut, canada, v. 1.0 (2007-2019). https://nordicana.cen.ulaval.ca/dpage.aspx?doi=45645CE-A24D883A6676492E.

Gauthier, G., Cadieux, M., Seyer, Y., and Therrien, J. (2020). Monitoring of avian predator reproduction on bylot island, nunavut, canada. https://nordicana.cen.ulaval.ca/dpage.aspx?doi=45591AW-F9B906CC647948E0.

Gauthier, G., Cadieux, M.-C., Berteaux, D., Bêty, J., Fauteux, D., Legagneux, P., Lévesque, E., and Gagnon, C.-A. (2024b). Long-term study of the tundra food web at a hotspot of arctic biodiversity, the bylot island field station. *Arctic Science*, 10(1):108–124.

Gauthier, G., Legagneux, P., Valiquette, M.-A., Cadieux, M.-C., and Therrien, J.-F. (2015). Diet and reproductive success of an arctic generalist predator: Interplay between variations in prey abundance, nest site location, and intraguild predation. *The Auk: Ornithological Advances*, 132(3):735–747.

Gill, F., Donsker, D., and Rasmussen, P. (2024). Ioc world bird list (v14. 2).

Goto, D., Dunlop, E. S., Young, J. D., and Jackson, D. A. (2020). Shifting trophic control

of fishery–ecosystem dynamics following biological invasions. *Ecological Applications*, 30(8):e02190.

Griffith, G. P., Hop, H., Vihtakari, M., Wold, A., Kalhagen, K., and Gabrielsen, G. W. (2019). Ecological resilience of arctic marine food webs to climate change. *Nature Climate Change*, 9(11):868–872.

Gruyer, N., Gauthier, G., and Berteaux, D. (2008). Cyclic dynamics of sympatric lemming populations on bylot island, nunavut, canada. *Canadian Journal of Zoology*, 86(8):910–917.

Hubbell, S. P. (2001). *The Unified Neutral Theory of Biodiversity and Biogeography*. Princeton University Press, Princeton.

Hutchison, C., Guichard, F., Legagneux, P., Gauthier, G., Bêty, J., Berteaux, D., Fauteux, D., and Gravel, D. (2020). Seasonal food webs with migrations: multi-season models reveal indirect species interactions in the canadian arctic tundra. *Philosophical Transactions of the Royal Society A*, 378(2181):20190354.

Ims, R., Ehrich, D., Forbes, B., Huntley, B., Walker, D., and Wookey, P. A. (2013). Terrestrial ecosystems (chapter 12). In Meltoffe, H., Josefson, A. B., and Payer, D., editors, *Arctic Biodiversity Assessment: Status and trends in Arctic biodiversity*, pages 385–440. Conservation of Arctic Flora and Fauna (CAFF).

Kahle, D. and Wickham, H. (2013). ggmap: Spatial visualization with ggplot2. *The R Journal*, 5(1):144–161.

Kemp, W., Harvey, S., and O'neill, K. (1990). Patterns of vegetation and grasshopper community composition. *Oecologia*, 83:299–308.

Klassen, R. (1993). *Quaternary geology and glacial history of Bylot Island, Northwest Territories*, volume 429. Geological Survey of Canada.

Krebs, C. J., Danell, K., Angerbjörn, A., Agrell, J., Berteaux, D., Bråthen, K. A., Danell, Ö., Erlinge, S., Fedorov, V., Fredga, K., Hjältén, J., Högstedt, G., Jónsdóttir, I. S., Kenney, A. J., Kjellén, N., Nordin, T., Roininen, H., Svensson, M., Tannerfeldt, M., and Wiklund, C. (2003). Terrestrial trophic dynamics in the canadian arctic. *Canadian journal of Zoology*, 81(5):827–843.

Lai, S., Bêty, J., and Berteaux, D. (2015). Spatio–temporal hotspots of satellite–tracked arctic foxes reveal a large detection range in a mammalian predator. *Movement ecology*, 3(37):1–10.

Lai, S., Bêty, J., and Berteaux, D. (2017). Movement tactics of a mobile predator in a meta-ecosystem with fluctuating resources: the arctic fox in the high arctic. *Oikos*, 126(7):937–947.

Lai, S., Warret Rodrigues, C., Gallant, D., Roth, J. D., and Berteaux, D. (2022). Red foxes at their northern edge: competition with the arctic fox and winter movements. *Journal of Mammalogy*, 103(3):586–597.

Lamarre, J.-F., Gauthier, G., Lanctot, R. B., Saalfeld, S. T., Love, O. P., Reed, E., Johnson, O. W., Liebezeit, J., McGuire, R., Russell, M., Nol, E., Koloski, L., Sanders, F., McKinnon, L., Smith, P. A., Flemming, S. A., Lecomte, N., Giroux, M. A., Bauer, S., Emmenegger, T., and Bêty, J. (2021). Timing of breeding site availability across the north-american arctic partly determines spring migration schedule in a long-distance neotropical migrant. *Frontiers in Ecology and Evolution*, 9:710007.

Lamarre, J.-F., Legagneux, P., Gauthier, G., Reed, E. T., and Bêty, J. (2017). Predator-mediated negative effects of overabundant snow geese on arctic-nesting shorebirds. *Ecosphere*, 8(5):e01788.

Léandri-Breton, D.-J., Lamarre, J.-F., and Bêty, J. (2019). Seasonal variation in migration strategies used to cross ecological barriers in a nearctic migrant wintering in africa. *Journal of Avian Biology*, 50(6):e02101.

Lecomte, N., Gauthier, G., and Giroux, J.-F. (2008). Breeding dispersal in a heterogeneous landscape: the influence of habitat and nesting success in greater snow geese. *Oecologia*, 155:33–41.

Legagneux, P., Gauthier, G., Berteaux, D., Bêty, J., Cadieux, M.-C., Bilodeau, F., Bolduc, E., McKinnon, L., Tarroux, A., Therrien, J.-F., Morisette, L., and Krebs, C. J. (2012). Disentangling trophic relationships in a high arctic tundra ecosystem through food web modeling. *Ecology*, 93(7):1707–1716.

Legagneux, P., Gauthier, G., Lecomte, N., Schmidt, N. M., Reid, D., Cadieux, M., Berteaux, D., Bety, J., Krebs, C., Ims, R., Yoccoz, N. G., Morrison, R. I. G., Leroux, S. J., Loreau, M., and Gravel, D. (2014). Arctic ecosystem structure and functioning shaped by climate and herbivore body size. *Nature Climate Change*, 4:379–383.

Lepage, D., Gauthier, G., and Reed, A. (1996). Breeding-site infidelity in greater snow geese: a consequence of constraints on laying date? *Canadian Journal of Zoology*, 74(10):1866–1875.

Lindenmayer, D. B., Likens, G. E., Andersen, A., Bowman, D., Bull, C. M., Burns, E.,

Dickman, C. R., Hoffmann, A. A., Keith, D. A., Liddell, M. J., Lowe, A. J., Metcalfe, D. J., Phinn, S. R., Russel-smith, J., Thurgate, N., and Wardle, G. M. (2012). Value of long-term ecological studies. *Austral Ecology*, 37(7):745–757.

Magurran, A. E. (2007). Species abundance distributions over time. *Ecology letters*, 10(5):347–354.

Magurran, A. E., Baillie, S. R., Buckland, S. T., Dick, J. M., Elston, D. A., Scott, E. M., Smith, R. I., Somerfield, P. J., and Watt, A. D. (2010). Long-term datasets in biodiversity research and monitoring: assessing change in ecological communities through time. *Trends in ecology & evolution*, 25(10):574–582.

Mazerolle, M. J. (2023). *AICcmodavg: Model selection and multimodel inference based on (Q)AIC(c)*. R package version 2.3.3.

McCann, K. S., Rasmussen, J., and Umbanhowar, J. (2005). The dynamics of spatially coupled food webs. *Ecology letters*, 8(5):513–523.

Miller, D. L., Rexstad, E., Thomas, L., Marshall, L., and Laake, J. L. (2019). Distance sampling in R. *Journal of Statistical Software*, 89(1):1–28.

Moisan, L., Gravel, D., Legagneux, P., Gauthier, G., Léandri-Breton, D.-J., Somveille, M., Therrien, J.-F., Lamarre, J.-F., and Bêty, J. (2023). Scaling migrations to communities: An empirical case of migration network in the arctic. *Frontiers in Ecology and Evolution*, 10:1077260.

Montgomerie, R. D., Cartar, R. V., McLaughlin, R. L., and Lyon, B. (1983). Birds of sarcpa lake, melville peninsula, northwest territories: breeding phenologies, densities and biogeography. *Arctic*, 36(1):65–75.

Neufeld, L. (2021). Comparing migration ecology among geographically distinct populations of Canada Geese (Branta canadensis) and Cackling Geese (Branta hutchinsii). Master's thesis, University of Manitoba.

Okey, T. A., Banks, S., Born, A. F., Bustamante, R. H., Calvopiña, M., Edgar, G. J., Espinoza, E., Fariña, J. M., Garske, L. E., Reck, G. K., Salazar, S., Shepherd, S., Toral-Granda, V., and Wallem, P. (2004). A trophic model of a galápagos subtidal rocky reef for evaluating fisheries and conservation strategies. *Ecological Modelling*, 172(2-4):383–401.

Parmelee, D. F., Stephens, H., and Schmidt, R. H. (1967). *The birds of southeastern Victoria Island and adjacent small islands*. Number 78. Queen's Printer.

Payer, D. C., Josefson, A. B., and Fjeldsa, J. (2013). Species diversity in the arctic (chapter 2). In Meltoffe, H., Josefson, A. B., and Payer, D., editors, *Arctic Biodiversity Assessment: Status and trends in Arctic biodiversity*, pages 67–77. Conservation of Arctic Flora and Fauna (CAFF).

Pebesma, E. J. et al. (2018). Simple features for r: standardized support for spatial vector data. *The R Journal*, 10(1):439–446.

Philippi, T. E., Dixon, P. M., and Taylor, B. E. (1998). Detecting trends in species composition. *Ecological applications*, 8(2):300–308.

Plagányi, É. E. (2007). *Models for an ecosystem approach to fisheries*. Food and Agriculture Organization of the united nations.

Reed, A., Hughes, R. J., and Boyd, H. (2002). Patterns of distribution and abundance of greater snow geese on bylot island, nunavut, canada 1983-1998. *Wildfowl*, 53:53–65.

Robillard, A., Gauthier, G., Therrien, J.-F., and Bêty, J. (2018). Wintering space use and site fidelity in a nomadic species, the snowy owl. *Journal of Avian Biology*, 49(5):jav–01707.

Royer-Boutin, P. (2015). Effets des cycles de lemmings sur le succès de nidification d'oiseaux différant par leur taille corporelle et leur comportement. Master's thesis, Université du Québec à Rimouski, Rimouski, Québec.

Seyer, Y., Gauthier, G., Bernatchez, L., and Therrien, J.-F. (2019). Sexing a monomorphic plumage seabird using morphometrics and assortative mating. *Waterbirds*, 42(4):380–392.

Seyer, Y., Gauthier, G., Fauteux, D., and Therrien, J.-F. (2020). Resource partitioning among avian predators of the arctic tundra. *Journal of Animal Ecology*, 89(12):2934–2945.

Snyder, K. and Tartowski, S. (2006). Multi-scale temporal variation in water availability: implications for vegetation dynamics in arid and semi-arid ecosystems. *Journal of Arid Environments*, 65(2):219–234.

Taylor, P. S. (1974). Summer population and food ecology of jaegers and snowy owls on bathurst island, nwt emphasizing the long-tailed jaeger. Master's thesis, University of Alberta, Edmonton, Alberta.

Therrien, J.-F., Gauthier, G., and Bêty, J. (2012). Survival and reproduction of adult snowy owls tracked by satellite. *The Journal of Wildlife Management*, 76(8):1562–1567.

Therrien, J.-F., Gauthier, G., Korpimäki, E., and Bêty, J. (2014). Predation pressure by avian predators suggests summer limitation of small-mammal populations in the canadian arctic. *Ecology*, 95(1):56–67.

Upham, N., Burgin, C., Widness, J., Liphardt, S., Parker, C., Becker, M., Rochon, I., Huckaby, D., and Zijlstra, J. (2024). Mammal diversity database.

Wickham, H. (2016). *ggplot2: Elegant Graphics for Data Analysis.* Springer-Verlag New York.

Wickham, H. (2023). *stringr: Simple, Consistent Wrappers for Common String Operations.* R package version 1.5.1.

Wickham, H., François, R., Henry, L., Müller, K., and Vaughan, D. (2023a). *dplyr: A Grammar of Data Manipulation.* R package version 1.1.4.

Wickham, H., Pedersen, T. L., and Seidel, D. (2023b). *scales: Scale Functions for Visualization.* R package version 1.3.0.

Wickham, H., Vaughan, D., and Girlich, M. (2024). *tidyr: Tidy Messy Data.* R package version 1.3.1.

Wilman, H., Belmaker, J., Simpson, J., de la Rosa, C., Rivadeneira, M. M., and Jetz, W. (2014). Eltontraits 1.0: Species-level foraging attributes of the world's birds and mammals: Ecological archives e095-178. *Ecology*, 95(7):2027–2027.
